# The small molecule PSSM0332 disassociates the CRL4A^DCAF8^ E3 ligase complex to decrease the ubiquitination of NcoR1 and inhibit the inflammatory response in a mouse sepsis-induced myocardial dysfunction model

**DOI:** 10.7150/ijbs.50186

**Published:** 2020-09-19

**Authors:** Qingyun Peng, Huifen Xu, Mingbing Xiao, Linhua Wang

**Affiliations:** 1 Department of Critical Care Medicine, Affiliated Hospital of Nantong University, Nantong 226001, Jiangsu, China.; 2 Department of Gastroenterology and Research Center of Clinical Medicine, Affiliated Hospital of Nantong University, Nantong 226001, Jiangsu, China.

**Keywords:** SIMD, CUL4A, RBX1, NcoR1, HMGB1, PSSM0332

## Abstract

Sepsis-induced myocardial dysfunction (SIMD) is a life-threatening complication caused by inflammation, but how it is initiated is still unclear. Several studies have shown that extracellular high mobility group box 1 (HMGB1), an important cytokine triggering inflammation, is overexpressed during the pathogenesis of SIMD, but the underlying mechanism regarding its overexpression is still unknown. Herein, we discovered that CUL4A (cullin 4A) assembled an E3 ligase complex with RBX1 (ring-box 1), DDB1 (DNA damage-binding protein 1), and DCAF8 (DDB1 and CUL4 associated factor 8), termed CRL4A^DCAF8^, which ubiquitinated and degraded NcoR1 (nuclear receptor corepressor 1) in an LPS-induced SIMD mouse model. The degradation of NcoR1 failed to form a complex with the SP1 transcription factor, leading to the upregulation of *HMGB1*. Mature HMGB1 functioned as an effector to induce the expression of proinflammatory cytokines, causing inflammation and resulting in SIMD pathology. Using an *in vitro* AlphaScreen technology, we identified three small molecules that could inhibit the CUL4A-RBX1 interaction. Of them, PSSM0332 showed the strongest ability to inhibit the ubiquitination of NcoR1, and its administration in SIMD mice exhibited promising effects on decreasing the inflammatory response. Collectively, our results reveal that the CRL4A^DCAF8^ E3 ligase is critical for the initiation of SIMD by regulating the expression of *HMGB1* and proinflammatory cytokines. Our results suggest that PSSM0332 is a promising candidate to inhibit the inflammatory response in the pathogenesis of SIMD, which will provide a new option for the therapy of SIMD.

## Introduction

Sepsis-induced myocardial dysfunction (SIMD) is a deadly symptom of sepsis caused by inflammation 1, 2. A few studies report that bacterial endotoxins (lipopolysaccharides, LPS), extracellular high mobility group box 1 (HMGB1) and inflammatory cytokines such as interleukin 1 beta (IL-1β) and tumor necrosis factor alpha (TNF-α) can trigger the pathogenesis of SIMD 1, 2. However, it is still obscure how these biological processes are initiated and what is the aberrantly expressed gene profile controlled by these stimuli in the pathological process of SIMD. The inflammatory signaling pathways have been well characterized in many diseases. Upon stimulation with LPS and HMGB1, their coreceptor TLR4 (Toll-like receptor 4) on the cell membrane is activated and recruits several intracellular effectors, including TIRAP (Toll/interleukin-1 receptor domain-containing adaptor protein), MyD88 (myeloid differentiation primary response gene 88), IRAK1 (IL1 receptor-associated kinase) and IRAK4, to form a complex 3-5. This complex initiates a signaling cascade consisting of TRAF6/TAK1/IKKs (TNF receptor-associated factor 6/transforming growth factor-β-activated kinase 1/IκB kinases), causing the phosphorylation of IκB and allowing the release of NF-κB (nuclear factor κB) from the IκB-NF-κB complex 3-5. The released NF-κB translocates from the cytoplasm to the nucleus, where it transactivates proinflammatory cytokine genes such as *IL1B*, *IL6*, *IL15* and *TNFA* 3-5. Emerging evidence has shown that expression of the *HMGB1* gene is significantly increased during sepsis in activated immune cells and necrotic tissues 6-8. However, it is still unknown how *HMGB1* is activated during this biological process.

In eukaryotes, the expression of genes is regulated by transcription factors (TFs) such as NF-κB, p53, AP1 (activator protein 1) and SP1 9. TFs recognize specific DNA sequences located in the promoters of genes to control chromatin and transcription 10. In addition to TFs, transcriptional regulators such as coactivators and corepressors also mediate gene expression 11-14. Biochemically, coactivators and corepressors are incapable of independent DNA binding, and they are recruited by TFs to assemble a complex 11-14. Coactivators coordinate with TFs to induce gene expression, and the well-known coactivators include histone acetyltransferases (HATs), such as p300, CBP (CREB-binding protein), KAT2A (lysine acetyltransferase 2A) and PCAF (p300/CBP-associated factor) 11-14. Conversely, corepressors function to repress gene transcription by activating histone deacetylation, and they mainly include CtBPs (C-terminal binding proteins) and NcoRs (nuclear receptor corepressors) 11-14. Mammalian genomes encode two NcoR members: NcoR1 and NcoR2 15, 16. Both of them have been shown to regulate gene expression by activating HDAC3 (histone deacetylase 3) through their deacetylase activation domain 15, 16.

The ubiquitin/proteasome system (UPS) is a conserved protein modification mechanism in eukaryotes and is implicated in many diseases, such as cancer, neurodegenerative diseases and inflammatory diseases 17-20. Three distinct enzymes, ubiquitin activating enzyme (E1), ubiquitin conjugating enzyme (E2) and ubiquitin ligase (E3), are involved in protein ubiquitination 17-20. With these enzymes, the ubiquitination reaction proceeds in three discrete steps: (a) an E1 enzyme utilizes ATP to activate ubiquitin and transfers it to an E2 enzyme; (b) an E2 enzyme interacts with a member of an E3 complex and transfers ubiquitin to a substrate protein; and (c) an activated E3 ligase ubiquitinates and degrades the substrate 17-20. Eukaryotic genomes encode more than 600 E3 ligases, which can modify thousands of substrates, allowing tremendous diversity in both substrates and biological processes 17-20. Based on the presence of protein domains and ubiquitin transfer patterns, E3 ligases can be classified into three major types: RING E3s, HECT (homologous to the E6AP carboxyl terminus) E3s and RBP (RING-between RING-RING) E3s 21, 22. RING E3s are the most abundant type of ligases, and they all contain a zinc-binding domain called RING 21, 22. Among RING E3s, cullin-RING ligases (CRLs) comprise the largest subfamily and are assembled by a RING-box (RBX) protein, a cullin scaffold, an adaptor protein and a substrate receptor 21, 22. Mammalian genomes encode 8 cullin members, CUL1, 2, 3, 4A, 4B, 5, 7 and 9. Although CUL4A and CUL4B are two highly conserved paralogs with over 80% amino acid identity, they do not show significant functional redundancy 21, 22. On a molecular level, both CUL4A and CUL4B assemble CRL4s with RBX1, DDB1 (DNA damage binding protein 1), and DCAFs (DDB1- and CUL4-associated factors) 21, 22. CRL4s ubiquitinate a variety of substrates, such as DDB2, cell cycle regulators p21 and p27, as well as tumor suppressors PTEN (phosphatase and tensin homolog) and ST7 (suppression of tumorigenicity 7) 23-25. The aberrant regulation of CRL4-dependent ubiquitination causes many diseases by affecting DNA damage and repair, cell death, and cell cycle progression 21-25. However, it is unknown whether cullins, especially CUL4s, are involved in the pathogenesis of SIMD.

To explore the aberrantly expressed gene profile in the pathogenesis of SIMD, we established an LPS-induced mouse model and performed a microarray analysis using SIMD heart tissues. We found that *CUL4A* was significantly overexpressed in SIMD heart tissues compared to controls. Further investigation revealed that CUL4A assembled an E3 ligase complex with RBX1, DDB1 and DCAF8. The CRL4A^DCAF8^ E3 ligase recognized NcoR1 as a substrate, and CUL4A overexpression caused the degradation of NcoR1. We also provide evidence that SP1 recruited NcoR1 to the promoter of *HMGB1*. The degradation of NcoR1 resulted in the upregulation of *HMGB1*, whose maturation and secretion to the extracellular space triggered TLR4/NF-κB signaling to induce the expression of inflammatory cytokine genes, thereby aggravating the inflammatory response and causing SIMD pathogenesis. Importantly, we also identified PSSM0332 could target the RBX1-CUL4A interaction and evaluated its effect on the ubiquitination of NcoR1, *HMGB1* expression and the inflammatory response.

## Materials and methods

### Establishment of an LPS-induced SIMD mouse model and tissue collection

As previously described 26, 27, an LPS-induced SIMD mouse model was established to mimic the initial clinical features of human SIMD. Accordingly, wild-type C57BL/6 mice (n=40) were divided into two groups (n=20 for each group) and then intraperitoneally injected with LPS (10 mg/kg) (Sigma-Aldrich, Shanghai, China, #L4391) or sterile PBS (phosphate-buffered saline) (Sigma-Aldrich, #806552) in a 25-μL volume. The injected mice were caged for another 12 h, followed by ultrasound detection to determine cardiac function. Blood samples and heart tissues were collected in accordance with an experimental protocol reviewed by the Ethics Committee of Nantong University Affiliated Hospital. The blood samples were stored in EDTA-coated tubes (BD, Franklin Lakes, NJ, USA, #367835). The heart tissues were immediately frozen in liquid nitrogen until RNA and protein extraction.

### Enzyme-linked immunosorbent assay (ELISA)

The serum concentrations of four proinflammatory cytokines (IL-1β, IL6, IL15 and TNF-α) and two anti-inflammatory cytokines (IL4 and IL13) were measured using ELISA in accordance with the manufacturer's guidelines. The ELISA kits were all purchased from Thermo Fisher Scientific company (Waltham, MA, USA), and their catalog numbers were as follows: #BMS6002 (IL-1β), #KMC0061 (IL6), #BMS6023 (IL15), #88732422 (TNF-α), #BMS613 (IL4), and #BMS6015 (IL13).

### Cell culture and transfection

The mouse macrophage cell line RAW264.7 (#TIB-71) was purchased from American Type Culture Collection (ATCC, VA, USA) and cultured in Dulbecco's modified Eagle's medium (DMEM) (Sigma-Aldrich, #D0819) supplemented with 10%, fetal bovine serum (FBS) (Sigma-Aldrich, #12003C) and 100 U/mL antibiotics (Sigma-Aldrich, #P4083) at 37°C with 5% CO_2_. For the transfection of overexpression plasmids, they were transfected into cells with Lipofectamine 3000 (Thermo Fisher Scientific, #L3000001) following the manufacturer's protocol. For the transfection of shRNAs, two independent shRNA lentiviral transduction particles of each genes were purchased from Sigma-Aldrich and their information was as follows: shCUL4A (#TRCN0000353106 and #TRCN0000012783), shCUL4B (#TRCN0000273707 and #TRCN0000273755), shNcoR1 (#TRCN0000096476 and #TRCN0000096477), shSP1 (#TRCN0000071603 and #TRCN0000071606), shc-MYC (#TRCN0000042515 and #TRCN0000042517), shNFYA (#TRCN0000331581 and (#TRCN0000084438), shRELA (#TRCN0000360656 and #TRCN0000360657), shNFKB1 (#TRCN0000235485 and #TRCN0000235486), and shSTAT4 (#TRCN0000235840 and #TRCN0000235843). These particles were individually transfected into RAW264.7 cells with FuGene 6 (Roche Diagnostics Corp., Indianapolis, IN, USA, #E2691) according to the manufacturer's method. Cells were then selected with puromycin (1 μg/mL) for 48 h and single puromycin-resistant cells were picked out for further culture, followed by determining mRNA and protein levels of the targets. The successful knockdown cells were applied to the required experiments.

### RNA isolation, microarray analysis and real-time quantitative PCR (RT-qPCR) analysis

The cultured cells and mouse heart tissues were harvested for RNA isolation with TRIzol reagent (Thermo Fisher Scientific, #15596026). Total RNA (1.0 μg) was subjected to microarray analysis with a mouse-specific kit (Agilent, Santa Clara, CA, USA, #G4846A) following the guidelines provided by the manufacturer. For the quantification of gene expression, total RNA (1.0 μg) was used for reverse transcription to generate first-strand cDNA with a kit (Thermo Fisher Scientific, #AB1453A). RT-qPCR analyses with a SYBR Green kit (Sigma-Aldrich, #QR0100) were performed to detect gene expression using the primers listed in Supplementary Table 1. The relative expression of individual genes was normalized to β-actin expression using the 2^-ΔΔCt^ method.

### Western blotting

Total protein extracts were isolated from cultured cells and heart tissues with RIPA lysis buffer (Thermo Fisher Scientific, #89900) supplemented with a protease inhibitor cocktail (Cell Signaling Technology, Shanghai, China; #5871). Equal amounts of proteins (50 μg) were resolved by a 10% SDS-PAGE gel and transferred onto a PVDF (polyvinylidene difluoride) membrane (Thermo Fisher Scientific, #PB9320) for immunodetection. After blocking in 5% milk for 1 h, membranes were probed with antibodies specific to CUL1 (Santa Cruz Biotechnology, Dallas, TX, USA, #sc-17775), CUL2 (#sc-166506), CUL3 (#sc-166054), CUL4A, CUL4B, CUL5 (#373822), CUL7 (#sc-53810), CUL9 (Invitrogen, Carlsbad, CA, USA, #PA5-20277), RBX1 (#sc-393640), DDB1 (#sc-137142), DCAF8 (Antibodies-online, Limerick, PA, USA, #ABIN6089940), NcoR1 (Sigma-Aldrich, #AV32479), SP1 (Sigma-Aldrich, #WH0006667M2), HMGB1 (Sigma-Aldrich, #H9539), NFYA (Sigma-Aldrich, #SAB4502001), c-MYC (#sc-40), RELA (Abcam, Cambridge, UK, #ab16502), NFKB1 (Abcam, #ab32360), STAT4 (Abcam, #ab235946), Flag (Sigma-Aldrich, #F3165), and GAPDH (Abcam, #ab8245) at 4°C overnight, followed by probing with peroxidase-labeled secondary antibodies at 23°C for 90 min. After rinsing five times with PBST buffer, proteins were visualized using an enhanced chemiluminescence substrate (Thermo Fisher Scientific, #32106).

### Immunoprecipitation (IP) and mass spectrometry

Equal weights of heart tissues from three control mice or three SIMD mice were mixed and homogenized with RIPA lysis buffer containing a protease inhibitor. Total cell extracts were centrifuged at 18,000 *g* for 15 min at 4°C. The supernatant fractions were immunoprecipitated using anti-DDB1 and anti-DCAF8 antibodies. The purified protein complexes were resolved by a 10% SDS-PAGE gel and then stained with a Pierce silver stain kit (Thermo Fisher Scientific, #24612). The stained protein bands were cut into ~0.5 mm small slices, followed by digestion with a Trypsin Kit (Thermo Fisher Scientific, #60109101) and mass spectrometry analysis according to a previous protocol 25.

### Coimmunoprecipitation (co-IP) and *in vitro* pulldown assays

The coding sequences of DDB1, CUL4A, RBX1, DCAF8 and NcoR1 were cloned into the pcDNA3-6×Myc and pcDNA3-2×Flag empty vectors. Different combinations of plasmids, pcDNA3-2×Flag + pcDNA3-6×Myc-CUL4A, pcDNA3-2×Flag + pcDNA3-6×Myc-DCAF8, pcDNA3-2×Flag + pcDNA3-6×Myc-RBX1, pcDNA3-2×Flag-DDB1 + pcDNA3-6×Myc-CUL4A, pcDNA3-2×Flag-DDB1 + pcDNA3-6×Myc-DCAF8, pcDNA3-2×Flag-DDB1 + pcDNA3-6×Myc-RBX1, pcDNA3-2×Flag + pcDNA3-6×Myc-DDB1, pcDNA3-2×Flag + pcDNA3-6×Myc-NcoR1, pcDNA3-2×Flag + pcDNA3-6×Myc-CUL4A, pcDNA3-2×Flag-DCAF8 + pcDNA3-6×Myc-DDB1, pcDNA3-2×Flag-DCAF8 + pcDNA3-6×Myc-NcoR1, or pcDNA3-2×Flag-DCAF8 + pcDNA3-6×Myc-CUL4A, were cotransfected into RAW264.7 cells. After incubation at 37°C for 48 h, cells were lysed and centrifuged. Proteins in the supernatant were incubated with anti-Flag agarose conjugate (Sigma-Aldrich, #A4596) or anti-Myc agarose conjugate (Sigma-Aldrich, #A7470). The immunoprecipitated proteins were washed 5 times with RIPA buffer, and protein interactions were determined by immunoblots. For the *in vitro* pulldown assay, pET28a-CUL4A (His-tag) and pGEX-6P1-RBX1 (GST-tag) were expressed in the *E. coli* DE3 strain. The purified His-CUL4A and GST-RBX1 were incubated at 4°C for 1 h, and the protein mixture was divided into two parts. One part was incubated with GST beads (Thermo Fisher Scientific, #20211), and the other part was incubated with Ni-NTA beads (Thermo Fisher Scientific, #R90110). After incubation at 4°C for 3 h, beads were washed with PBS buffer 5 times, and the resulting proteins were resolved in a 10% SDS-PAGE gel, followed by Coomassie blue staining.

### Immunohistochemistry (IHC) staining

Three paired heart tissues from untreated mice (Control), SIMD mice and SIMD mice treated with small molecules were fixed in 10% formalin-PBS buffer for 24 h, followed by embedding in paraffin and sectioning to ~ 5 μm thickness. The slides were stained with anti-CUL4A, anti-RBX1, anti-DCAF8, anti-NcoR1, and anti-HMGB1 following a previous protocol 26, 27.

### Chromatin immunoprecipitation (ChIP) assay

ChIP assays were carried out as described previously 28. In brief, cells were crosslinked with 1% formaldehyde-PBS for 10 min at room temperature. After stopping the reaction with 125 mM glycine for 5 min, cells were rinsed twice with PBS buffer and then subjected to ChIP assay using a kit (Sigma-Aldrich, #17295) following the protocol provided by the manufacturer. The antibodies used for immunoprecipitation were anti-NcoR1 and anti-SP1. Mouse IgG was used as a negative control. The enriched DNA samples were diluted 10-fold and then subjected to RT-qPCR analyses with the following primers: forward, 5'-TGCAGACTAGGCTTCTGGG-3'; and reverse, 5'-TGGGATGTGCGGCCCGTGCT-3'. The relative occupancies of NcoR1 and SP1 on the promoter of *HMGB1* were calculated using the 2^-ΔΔCt^ method in which ΔCt=Ct_ouput_-Ct_input_.

### Screening small molecules in an AlphaScreen system

AlphaScreen was performed to identify small molecules that disrupted the CUL4A-RBX1 interaction using a previous protocol 29. In brief, a His-CUL4A/GST-RBX1 concentration matrix was set up in 30 μL of buffer: 7.5 μL of each protein, 5 μL glutathione donor beads and 5 μL nickel chelate acceptor beads from an AlphaScreen kit (PerkinElmer, Waltham, MA, USA, #6760603M), and 5 μL plant sourced small molecules (PSMM) (n=2500). The mixture was incubated at 25°C for 2 h, and then the plates were read in an Envision Multilabel Reader (PerkinElmer, #2105-0010). The AlphaScreen signal in a well only containing His-CUL4A/GST-RBX1, donor beads and acceptor beads but without adding a small molecule was set as a control, and the small molecules that decreased the signal less than 4000 were selected as candidates. A secondary round of AlphaScreen was performed to verify the inhibitory efficiency of the candidate small molecules.

### Administration of small molecules in SIMD mice

Similar weight (~22 g) SIMD mice (n=96) were selected to evaluate the effects of small molecules. The DMSO-dissolved small molecules, including PSSM0332, PSSM0856 and PSSM1437, were diluted in 50 μL PBS buffer to a final concentration of 4 μM. Small molecules were intraperitoneally injected into mice (n=24 for each group) at a 50 mg/kg dosage. After 24 h of injection, blood and heart tissue samples were collected.

### *In vivo* ubiquitination assay

The* in vivo* ubiquitination assay was performed as described previously 25. Briefly, both pcDNA3-2×Flag-NcoR1 and HA-ubiquitin plasmids were cotransfected into RAW264.7 (control), CUL4A-knockdown (KD), CUL4B-KD, and DCAF8-KD cells, and the transfected cells were incubated at 37°C for 48 h. Moreover, the control cells coexpressing pcDNA3-2×Flag-NcoR1 and HA-ubiquitin were also treated with 4 μM small molecules, PSSM0332, PSSM0856 and PSSM1437, for 6 h. Cells were harvested and lysed in RIPA buffer containing protease inhibitor, followed by IP with anti-Flag agarose. The enriched Flag-NcoR1 protein complexes were resolved in an 8% SDS-PAGE gel, and the ubiquitination of NcoR1 was determined using an anti-HA antibody.

### Statistical analysis

Except for microarray assay, the other experiments in this study were independently replicated three times. Each independent replicate contained triplicates. Data were analyzed using SPSS 22.0 software and were presented as the mean ± standard deviation (SD). The significance levels were set at *P* < 0.05 (*), *P* < 0.01 (**) and *P* < 0.001 (***).

## Results

### CUL4A was overexpressed in SIMD mice

**Figure 1:**
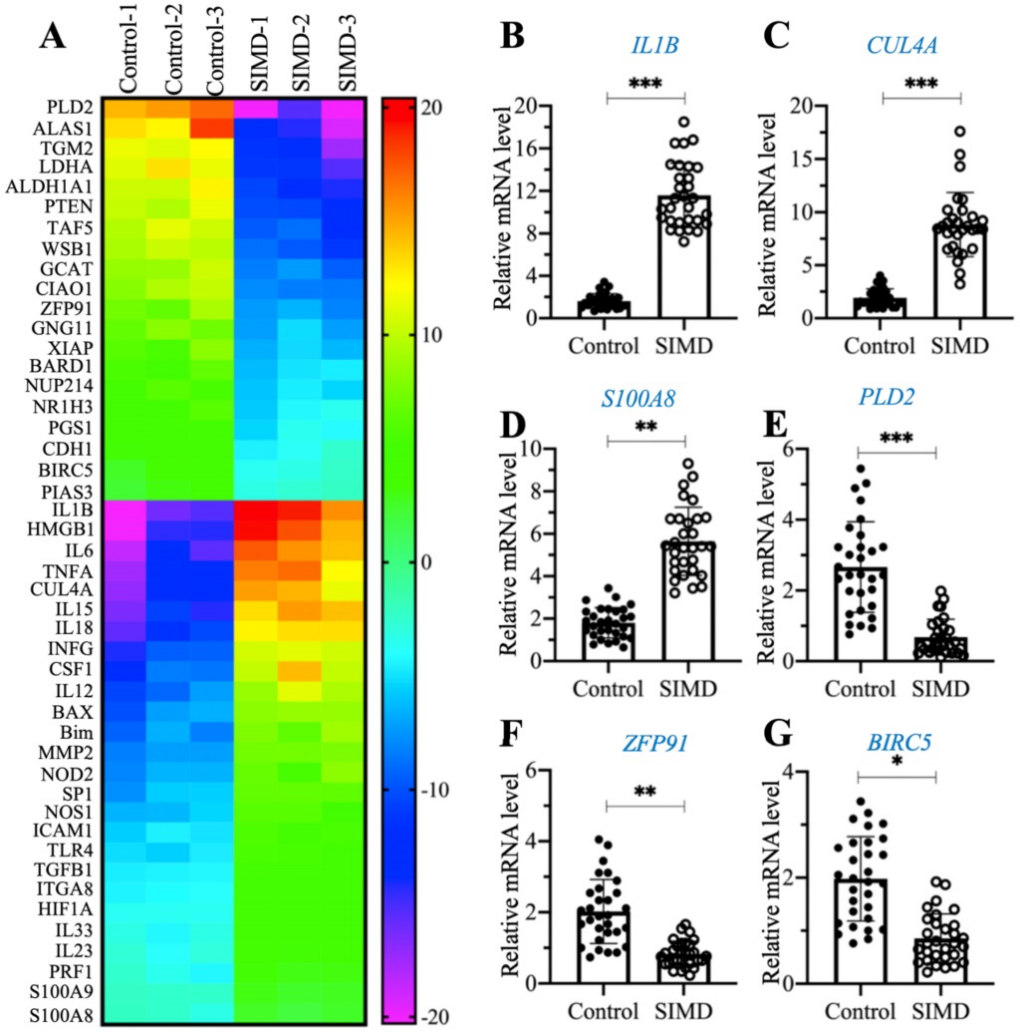
** The identification and verification of aberrantly expressed genes in SIMD heart tissues.** (**A**) Heat map of differentially expressed genes identified in SIMD heart tissues. Total RNA samples from three independent heart tissues of control mice and SIMD mice were used for microarray analysis. The aberrantly expressed genes that showed converse expression patterns between controls and SIMD mice were selected and presented in a heat map. (**B-G**) RT-qPCR results. Total RNA samples isolated from heart tissues of controls (n=24) and SIMD mice (n=24) were used to detect the mRNA levels of three overexpressed genes, *IL1B* (B), *CUL4A* (C), and *S100A8* (D), and three downregulated genes, *PLD2* (E), *ZFP91* (F), and *BIRC5* (G). * *P* < 0.05, ** *P* < 0.01 and *** *P* < 0.001.

To explore the dysregulated genes by whole genome-wide transcript profiling during the pathogenesis of SIMD, we established an LPS-induced SIMD mouse model (Supplementary Figure 1A). Previous studies have shown that inflammation is a common symptom in SIMD patients and animal models 1, 2. Similarly, we also validated the inflammatory status in SIMD mice by measuring the serum concentrations of proinflammatory cytokines. As shown in Supplementary Figures 1B-1E, the circulating concentrations of IL-1β, IL6, IL15 and TNF-α were significantly increased in SIMD mice compared to controls. However, the serum concentrations of two anti-inflammatory cytokines, IL4 and IL13, were not dramatically changed in the two groups of mice (Supplementary Figures 1F and 1G). Using total RNA from heart tissues of three representative control and SIMD mice, we performed a microarray analysis to identify the differentially expressed genes. After normalization, we found 46 genes that were consistently upregulated or downregulated in all three SIMD samples (Figure 1A and Supplementary Table 2). The overexpressed genes in SIMD mice included a variety of proinflammatory genes, such as *IL1B*, *IL6*, *IL15*, *IL18* and *TNFA*. In addition, we also found that *HMGB1*, *CUL4A*, *TLR4*, *SP1*, two pro-apoptotic genes, *BAX* and *Bim*, and two calcium binding genes, *S100A8* and *S100A9,* were significantly overexpressed in SIMD mice (Figure 1A and Supplementary Table 2). To verify the accuracy of our microarray results, we randomly selected three upregulated genes (*IL1B*, *CUL4A* and *S100A8*) and three downregulated genes [*PLD2* (phospholipase D2], *ZFP91* (zinc finger protein 91) and *BIRC5* (baculoviral IAP repeat-containing protein 5)] to examine their expression levels in SIMD and control mice (n=24). The RT-qPCR results showed that the expression levels of these six genes were consistent with the microarray results (Figures 1B-1G). The expression levels of *IL1B*, *CUL4A* and *S100A8* were increased ~5.8-fold, 5.1-fold and 2.5-fold, respectively (Figures 1B-D). In contrast, the expression levels of *PLD2*, *ZFP91* and *BIRC5* were decreased ~3.8-fold, 2-fold and 1.5-fold, respectively (Figures 1E-G).

Among these differentially expressed genes, *CUL4A* drew our attention due to its important role in the assembly of E3 ligases and protein ubiquitination. Given that the cullin gene family has 8 members, we next aimed to determine the specificity of *CUL4A* overexpression in the pathogenesis of SIMD. Using the same 24 paired RNA samples from control mice and SIMD mice; we performed RT-qPCR analyses to examine the mRNA levels of the other 7 cullin genes. As shown in Supplementary Figures 2A-2G, we did not observe a significant change in the mRNA levels of *CUL1*, *CUL2*, *CUL3*, *CUL4B*, *CUL5*, *CUL7* or *CUL9*. Moreover, we also examined the protein levels of these cullin members in the same heart tissues used for the microarray analysis. Consistent with the mRNA results, we also observed that only CUL4A but not the other cullin proteins were increased in SIMD heart tissues in comparison to controls (Supplementary Figure 2H). The specific overexpression of *CUL4A* suggested that it might have a unique role in the regulation of protein ubiquitination during the SIMD pathological process.

### CUL4A assembled an E3 ligase complex with RBX1, DDB1 and DCAF8

**Figure 2:**
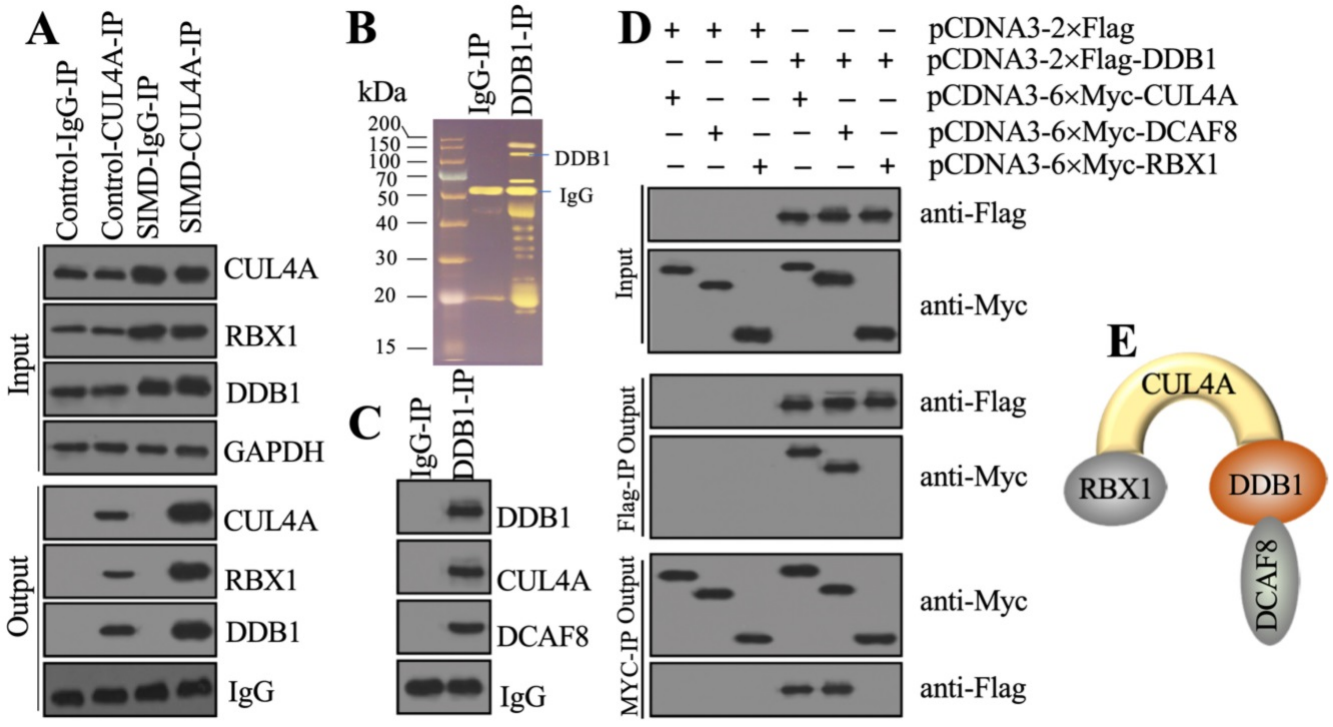
** CUL4A formed a complex with RBX1, DDB1 and DCAF8 *in vitro* and *in vivo*.** (**A**) *In vivo* IP results. Equal weights of heart tissues from three control or SIMD mice were mixed and then lysed in RIPA buffer to isolate total proteins. Total cell extracts were subjected to IP assay using anti-CUL4A-coupled protein A beads. The input and output proteins were used for immunoblotting to examine the protein levels of CUL4A, RBX1 and DDB1. GAPDH and IgG were the loading controls of input and output, respectively. (**B**) DDB1-associated proteins* in vivo*. Equal weights of SIMD heart tissues (n=3) were mixed together and lysed in RIPA buffer, followed by IP with IgG (negative control) or anti-DDB1-coupled protein A beads. The purified DDB1-associated protein complex was stained by a silver staining kit. The DDB1 and IgG bands were indicated. (**C**) DDB1 could pull down DCAF8 *in vivo*. The IP products used in (B) were applied to immunoblots to detect the protein levels of DDB1, CUL4A and DCAF8. IgG was used as a loading control. (**D**) Co-IP results. Cells were cotransfected with the plasmid combinations as indicated in the figure, followed by co-IP analyses using anti-Myc-agarose or anti-Flag-agarose beads. The input and output proteins were used to detect protein levels with anti-Flag and anti-Myc antibodies. (**E**) A representative model of the RBX1-CUL4A-DDB1-DCAF8 protein complex.

Previous studies have shown that CUL4A functions as a scaffold to recruit both RBX1 and DDB1 to assemble a complex 21, 22, 25. To determine whether this complex was also assembled in the development of SIMD, we performed *in vivo* IP assays in heart tissues from control and SIMD mice using CUL4A-coupled protein A beads. The immunoblot results showed that both RBX1 and DDB1 could be pulled down by CUL4A (Figure 2A). Importantly, SIMD-CUL4A could pull down much higher levels of both RBX1 and DDB1 (Figure 2A). Based on the knowledge of CRL4 E3 ligase assembly 21, 22, a DCAF protein was still lacking. To identify the DCAF protein associated with the RBX1-CUL4A-DDB1 complex, we performed an IP assay using anti-DDB1-coupled protein A beads (Figure 2B), followed by mass spectrometry analysis. Among the list of candidate interacting proteins, we only found DCAF8 (Supplementary Table 3). Using the same IP product as for the mass spectrometry analysis, we performed immunoblots to examine the *in vivo* association of DCAF8 with the RBX1-CUL4A-DDB1 complex. As expected, we found that DDB1 could pull down both CUL4A and DCAF8 *in vivo* (Figure 2C). We then performed co-IP assays to verify the direct interactions of DDB1-CUL4A, DDB1-DCAF8 and DDB1-RBX1. The co-IP assay results showed that DDB1 could directly interact with both CUL4A and DCAF8 but not RBX1 (Figure 2D). These results suggested that RBX1, CUL4A, DDB1 and DCAF8 could assemble a CRL4A^DCAF8^ E3 ligase complex in which CUL4A functioned as a linker to bind both RBX1 and DDB1, and DDB1 further recruited DCAF8 (Figure 2E).

### The CRL4A^DCAF8^ E3 ligase complex ubiquitinated and degraded NcoR1 in the development of SIMD

**Figure 3:**
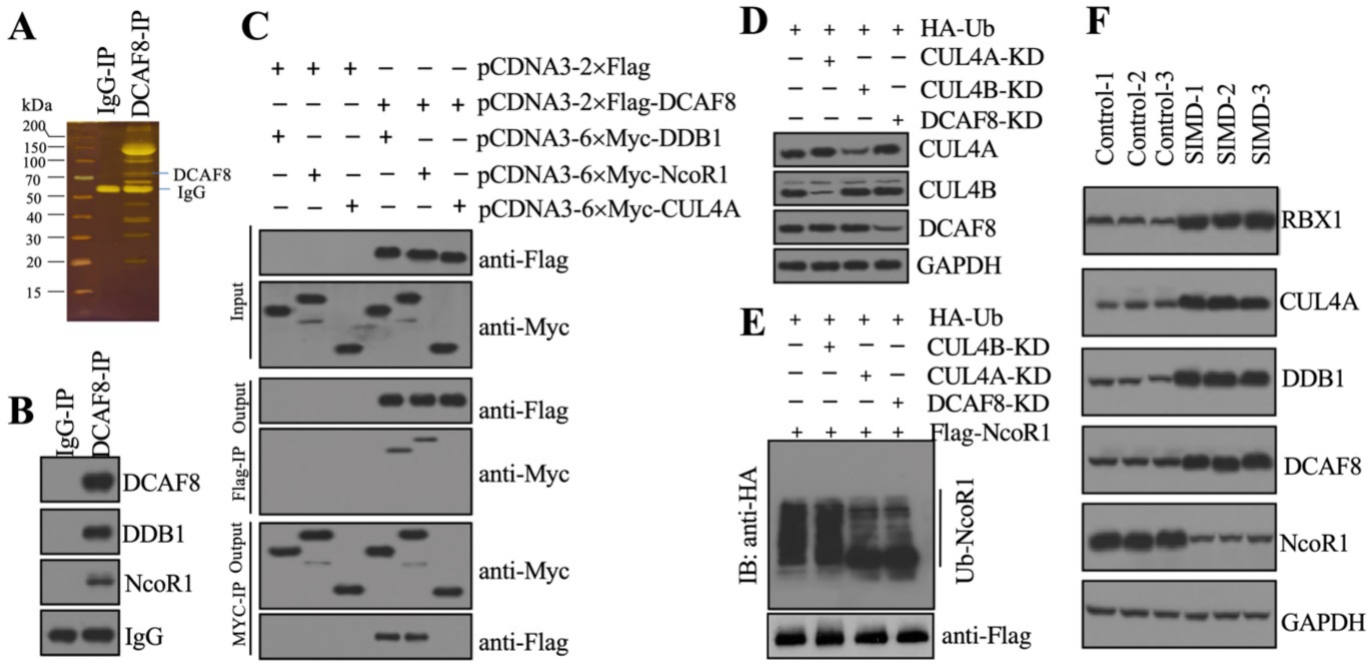
** CRL4A^DCAF8^ recognized NcoR1 as a substrate *in vivo*.** (**A**) DCAF8-associated proteins* in vivo*. Equal weights of hear tissues (n=3) were mixed together and lysed in RIPA buffer, followed by IP with IgG (negative control) and anti-DCAF8-coupled protein A beads. The purified DCAF8-associated protein complex was stained by a silver staining kit. The DCAF8 and IgG bands were indicated. (**B**) DCAF8 could pull down NcoR1 *in vivo*. The IP products used in (A) were applied to immunoblots to detect the protein levels of DCAF8, DDB1 and DCAF8. IgG was used as a loading control. (**C**) DCAF8 could pull down both DDB1 and NcoR1 *in vitro*. Cells were cotransfected with the plasmid combinations as indicated in the figure, followed by co-IP analyses using anti-Myc-agarose or anti-Flag-agarose beads. The input and output proteins were used to detect protein levels with anti-Flag and anti-Myc antibodies. (**D**) The protein levels of CUL4A/4B and DCAF8. The Control, CUL4A-KD, CUL4B-KD and DCAF8-KD cells coexpressing pcDNA3-2×Flag-NcoR1 and HA-ubiquitin were used for immunoblotting to examine the protein levels of CUL4A, CUL4B and DCAF8. GAPDH was used as a loading control. (**E**) The *in vivo* ubiquitination of NcoR1. Cells used in (D) were immunoprecipitated with an anti-Flag-agarose, and the ubiquitination of NcoR1 was detected using an anti-HA antibody. ^Flag^NcoR1 was used as a loading control. (**F**) The protein levels of CRL4A^DCAF8^ components and NcoR1 in SIMD heart tissues. Total cell extracts from three independent heart tissues of control and SIMD mice were used for western blotting to examine the protein levels of RBX1, CUL4A, DDB1, DCAF8 and NcoR1. GAPDH was used as a loading control.

To identify the substrate of CRL4A^DCAF8^ E3 ligase, we performed an IP assay using the anti-DCAF8-coupled protein A beads and the same cell extracts as in Figure 2B (Figure 3A). Following mass spectrometry analysis, we found an interesting protein, NcoR1, in the list of candidate interacting proteins (Supplementary Table 4). Using the same IP product as in Figure 3A, we performed immunoblotting to examine the *in vivo* association of DCAF8 with NcoR1. The results showed that NcoR1 could be pulled down by DCAF8 *in vivo* (Figure 3B). Similarly, we also performed co-IP assays to examine the direct interaction between DCAF8 and NcoR1 using the DCAF8-DDB1 interaction as a positive control. Our results indicated that DCAF8 could interact with NcoR1 (Figure 3C). Due to the difficulty of expressing the full length of NcoR1 in *E. coli* DE3 cells, we could not examine the ubiquitination of NcoR1 by an *in vitro* assay. Instead, we performed an *in vivo* analysis in cells coexpressing pcDNA3-2×Flag-NcoR1 and pcDNA3-2×HA-Ubiquitin under the conditions of silencing *CUL4A*, *CUL4B* or *DCAF8* (Figure 3D). Our data indicated that knockdown of both *CUL4A* and *DCAF8* but not *CUL4B* significantly decreased the NcoR1 ubiquitination level (Figure 3E). These results suggested that NcoR1 was a substrate of the CRL4A^DCAF8^ E3 ligase complex. To further confirm this conclusion, we also measured the protein levels of CRL4A^DCAF8^ members and NcoR1 in three paired heart tissues from control and SIMD mice. Our data showed that the components of the CRL4A^DCAF8^ complex were significantly increased in SIMD tissues compared to controls (Figure 3F). Conversely, the protein level of NcoR1 in SIMD heart tissues was dramatically decreased (Figure 3F). Thus, we concluded that NcoR1 was a substrate of the CRL4A^DCAF8^ E3 ligase complex and that it was ubiquitinated and degraded during the pathogenesis of SIMD.

### The expression of *HMGB1* was negatively associated with NcoR1

**Figure 4:**
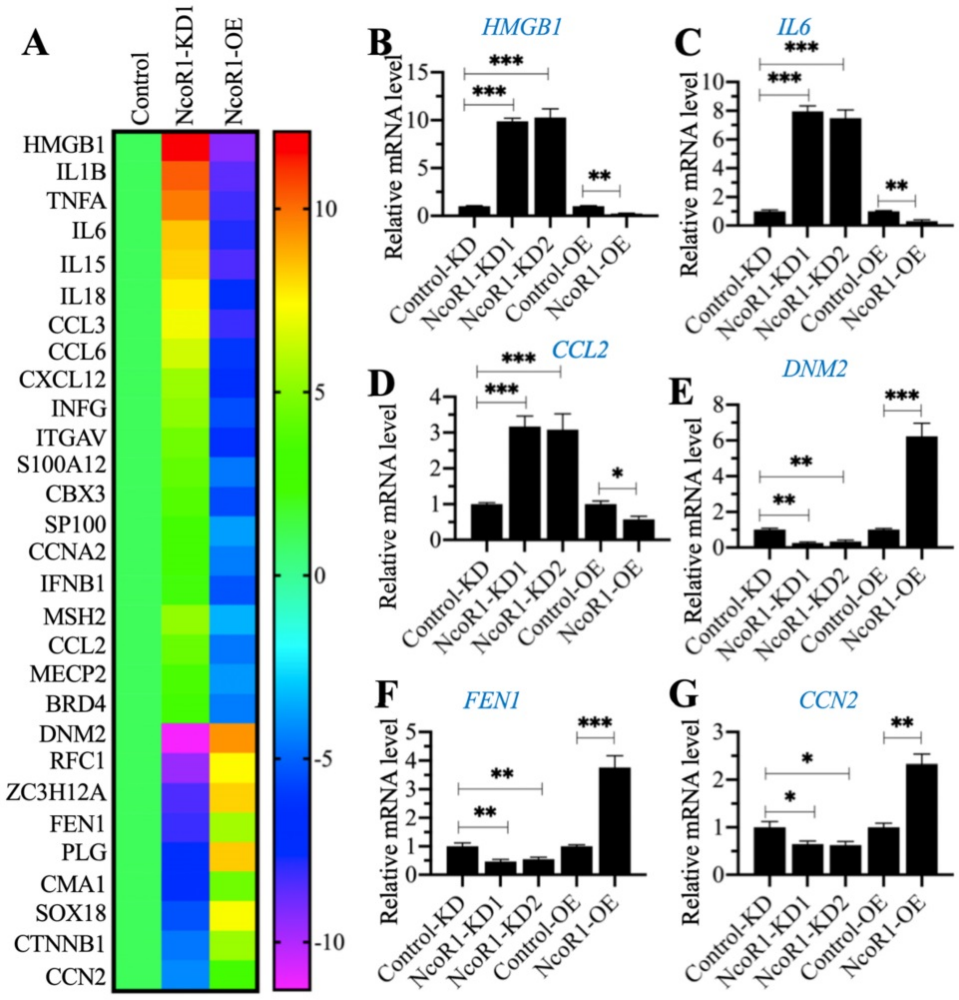
** The identification and verification of NcoR1-dependent genes.** (**A**) The heat map of differentially expressed genes dependent on *NcoR1*. Total RNA samples from RAW246.7 (Control), NcoR1-KD1 and NcoR1-OE cells were used for microarray analysis. The aberrantly expressed genes that showed converse expression patterns in NcoR1-KD1 and NcoR1-OE cells were selected and presented in a heat map. (**B-G**) RT-qPCR results. Total RNA samples isolated from Control-KD, NcoR1-KD (#1 and #2), Control-OE, and NcoR1-OE cells were used to detect the mRNA levels of six genes, *HMGB1* (B), *IL6* (C), *CCL2* (D), *DNM2* (E), *FEN1* (F), and *CCN2* (G). * *P* < 0.05, ** *P* < 0.01 and *** *P* < 0.001.

Given that NcoR1 is a transcriptional corepressor, we next aimed to identify the downstream target genes of NcoR1 by whole genome-wide transcript profiling. Accordingly, we generated two independent NcoR1-KD (#1 and #2) and one NcoR1-OE cell lines and verified the successful knockdown or overexpression of *NcoR1* in these cells (Supplementary Figure 3). Using total RNA from RAW246.7 (Control), NcoR1-KD1 and NcoR1-OE cells, we performed a microarray analysis to identify the NcoR1-dependent genes. After normalization, we found 29 genes that were conversely expressed in NcoR1-KD1 and NcoR1-OE cells (Figure 4A and Supplementary Table 5). We randomly selected three upregulated genes [*HMGB1*, *IL6* and *CCL2* (C-C motif chemokine ligand 2)] and three downregulated genes [*DNM2* (Dynamin 2], *FEN1* (flap structure-specific endonuclease 1) and *CCN2* (cellular communication network factor 2)] to examine their expression levels. Consistent with the microarray results, we also observed that the expression levels of *HMGB1*, *IL6* and *CCL2* were increased in NcoR1-KD cells but decreased in NcoR1-OE cells (Figures 4B-D). Conversely, the expression levels of *DNM2*, *FEN1* and *CCN2* were decreased in NcoR1-KD cells but increased in NcoR1-OE cells (Figures 4E-G). Comparing the gene lists in Figure 1A and Figure 4A, we found several overlapping genes, including *HMGB1*, *IL1B*, *IL6*, *IL15*, *IL18*, *TNFA*, and IFNG (interferon gamma) (Supplementary Tables 2 and 5). Using the same RNA samples as Figure 1B, we measured the expression of *HMGB1* in SIMD tissues and found it was also significantly increased (Supplementary Figure 4). Except for *HMGB1*, the other genes were targets of NF-κB, and TLR4/NF-κB signaling is activated during the development of SIMD. Moreover, HMGB1 is a well-known cytokine that triggers TLR4/NF-κB signaling in inflammation, suggesting that the overexpression of *HMGB1* might be the basic reason for the inflammatory response.

### NcoR1 interacted with SP1 to negatively control *HMGB1* expression

**Figure 5:**
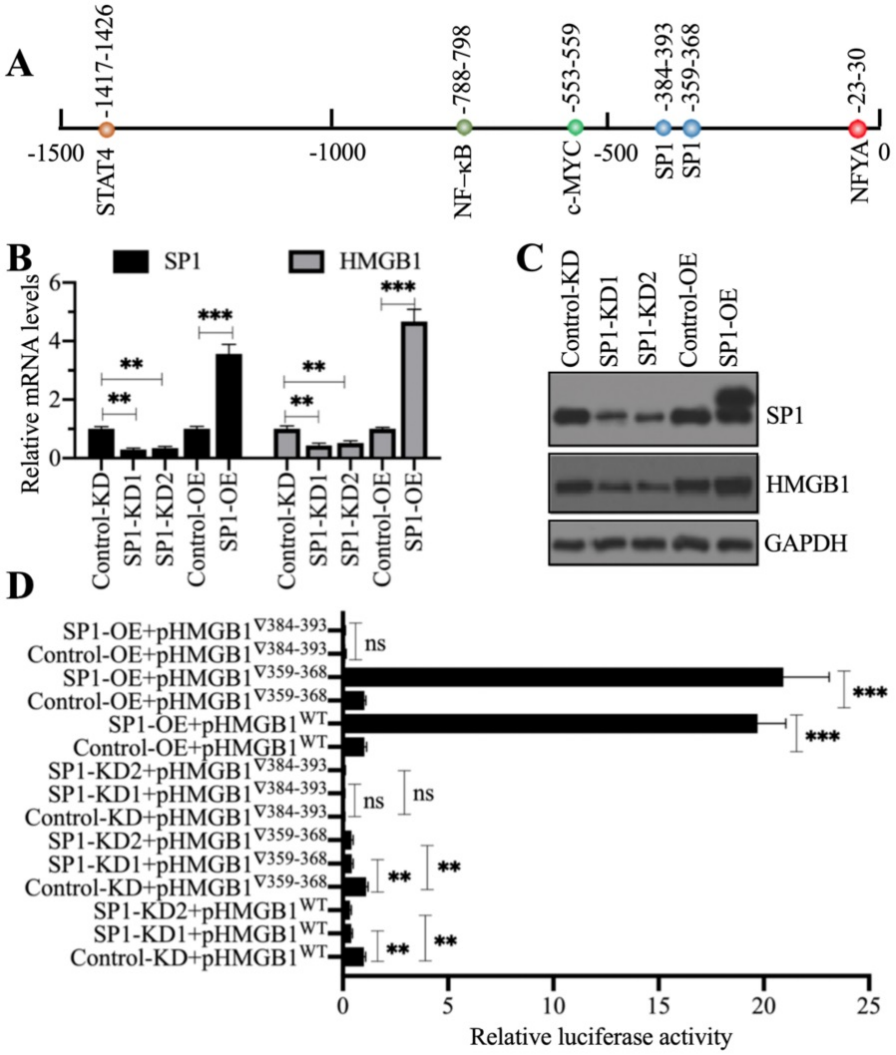
** SP1 specifically regulated the expression of *HMGB1*.** (**A**) The predicted TF binding sites in the promoter of *HMGB1*. A 1500-bp fragment of the *HMGB1* promoter was selected to predict the potential TF binding sites. One NFYA, two SP1, one c-MYC, one NF-κB, and one STAT4 binding site were identified, and their positions are indicated. (**B**) The relative mRNA levels of *SP1* and *HMGB1*. Total RNA from Control-KD, SP1-KD (#1 and #2), Control-OE, and SP1-OE cells was used to detect the mRNA levels of *SP1* and *HMGB1*. ** *P* < 0.01 and *** *P* < 0.001. (**C**) The protein levels of SP1 and HMGB1. Total cell extracts from cells used in (B) were subjected to immunoblotting to examine the protein levels of SP1 and HMGB1. GAPDH was used as a loading control. (**D**) The relative luciferase activity. Cells coexpressing pGL4.26-pHMGB1^WT^ (or pGL4.26-pHMGB1^▽359-368^ or pGL4.26-pHMGB1^▽384-393^) and Renilla into were subjected to a dual-luciferase reporter assay. ** *P* < 0.01 and *** *P* < 0.001. ns represents no significant difference.

Our above results showed that NcoR1 was a negative regulator of *HMGB1*. Given that NcoR1 is a corepressor and not a TF, we next sought to identify the TF associated with NcoR1 in the regulation of *HMGB1* expression. For this purpose, we predicted the TF binding sites in a 1500-bp fragment of the *HMGB1* promoter using CiiiDER software. We found five TF binding sites, an NFYA (nuclear transcription factor Y subunit alpha) site, two SP1 sites, one c-MYC site, one NF-κB site and one STAT4 site (Figure 5A). To explore which TF controlled the expression of *HMGB1*, we generated two independent knockdown cell lines and one overexpression cell line of these TFs. We found that only knockdown or overexpression of SP1 could change the expression of *HMGB1* (Figures 5B and 5C), while the knockdown or overexpression of *NFYA* (Supplementary Figures 5A and 5B), *c-MYC* (Supplementary Figures 5C and 5D), *RELA* (Supplementary Figures 5E and 5F), *NFKB1* (Supplementary Figures 5G and 5H), and *STAT4* (Supplementary Figures 5I and 5J) did not change the expression of *HMGB1*. These results suggested that only SP1 could positively control the expression of *HMGB1*. Because we found two SP1 binding sites [-359-(-)368 and -384-(-)393] in the promoter of *HMGB1*, we next determined the requirement for these two sites for the regulation of *HMGB1*. Accordingly, we constructed luciferase vectors containing the wild-type (WT) *HMGB1* promoter or deletions of the two SP1 binding sites. These vectors were cotransfected into Control-KD, SP1-KD1, SP1-KD2, Control-OE, and SP1-OE cells with the Renilla luciferase vector (internal control). The dual-luciferase reporter assay results indicated that the construct with deletion of the -384-(-)393 site failed to respond to SP1 knockdown and overexpression (Figure 5D). However, we did not observe a significant change in luciferase activity between the WT and -359-(-)368 site mutant (Figure 5D). These results suggested that the -384-(-)393 site but not the -359-(-)368 site was required for the binding of SP1. Importantly, SP1 might form a complex with NcoR1 to regulate the expression of *HMGB1*.

**Figure 6:**
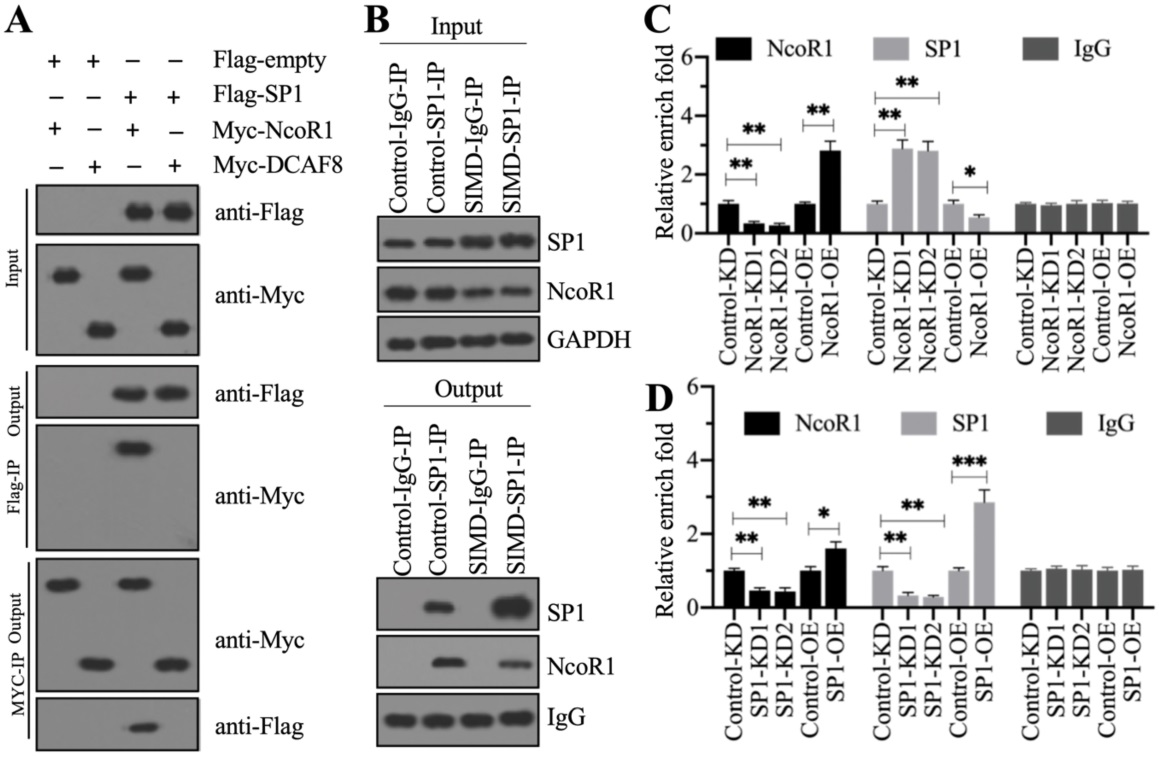
** NcoR1 directly interacted with SP1 to suppress its occupancy on the promoter of *HMGB1*.** (**A**) SP1 could pull down NcoR1 *in vitro*. Cells were cotransfected with the plasmid combinations as indicated in the figure, followed by co-IP analyses using anti-Myc-agarose or anti-Flag-agarose beads. The input and output proteins were used to detect protein levels with anti-Flag and anti-Myc antibodies. (**B**) SP1 could pull down NcoR1 *in vivo*. Equal weights of heart tissues from three control or SIMD mice were mixed and then lysed in RIPA buffer to isolate total proteins. Total cell extracts were subjected to IP assays using IgG and anti-SP1-coupled protein A beads. The input and output proteins were used for immunoblotting to examine the protein levels of SP1 and NcoR1. GAPDH and IgG were the loading controls of input and output, respectively. (**C**) The relative occupancies of NcoR1 and SP1 on the promoter of *HMGB1* in NcoR1-KD and NcoR1-OE cells. Cells were subjected to ChIP assays with anti-NcoR1, anti-SP1, or IgG. The purified DNA samples were subjected to RT-qPCR analyses to detect the occupancies of NcoR1 and SP1 on the promoter of *HMGB1*. * *P* < 0.05 and ** *P* < 0.01. (**D**) The relative occupancies of NcoR1 and SP1 on the promoter of *HMGB1* in SP1-KD and SP1-OE cells. Cells were subjected to ChIP assays with anti-NcoR1, anti-SP1, or IgG. The purified DNA samples were subjected to RT-qPCR analyses to detect the occupancies of NcoR1 and SP1 on the promoter of *HMGB1*. * *P* < 0.05, ** *P* < 0.01 and *** *P* < 0.001.

To determine whether NcoR1 could interact with SP1, we performed *in vitro* co-IP assays and *in vivo* pulldown assays. By co-IP assays, we found that ^Flag^SP1 could pull down ^Myc^NcoR1 but not ^Myc^DCAF8, and ^Myc^NcoR1 but not ^Myc^DCAF8 could pull down ^Flag^SP1 (Figure 6A). Using a mixture of heart tissues from control (n=3) and SIMD (n=3) mice, we carried out *in vivo* IP assays using the anti-SP1-coupled protein A beads. We found that SP1 could pull down NcoR1 in both control and SIMD tissues (Figure 6B). These results suggested that SP1 could interact with NcoR1 *in vitro* and *in vivo*. To further determine whether the SP1-NcoR1 complex could dock on the promoter of *HMGB1* to control its expression, we performed ChIP assays in two groups of cells, group I (Control-KD, NcoR1-KD1, NcoR1-KD2, Control-OE, and NcoR1-OE cells) and group II (Control-KD, SP1-KD1, SP1-KD2, Control-OE, and SP1-OE cells), using anti-NcoR1 and anti-SP1 antibodies, respectively. The ChIP results in group I cells indicated that knockdown of NcoR1 increased the occupancy of SP1 on the promoter of *HMGB1* (Figure 6C). However, the occupancy of NcoR1 was decreased with the knockdown of SP1 in group II cells (Figure 6D). These results suggested that SP1 primarily docked on the promoter of *HMGB1*, and it further recruited NcoR1 to negatively regulate the expression of *HMGB1*.

HMGB1 is a cytokine that triggers the activation of NF-κB and its downstream proinflammatory cytokine genes 4. Thus, we next aimed to evaluate the effects of the CRL4A^DCAF8^ components, NcoR1 and SP1 on the expression of proinflammatory cytokine genes. For this purpose, we generated CUL4A-KD (#1 and #2), CUL4A-OE, RBX1-KD (#1 and #2), RBX1-OE, NcoR1-KD (#1 and #2), NcoR1-OE, SP1-KD (#1 and #2) and SP1-OE cells and then examined the mRNA levels of three representative proinflammatory cytokine genes (*IL1B*, *IL6* and *TNFA*). Our data indicated that the knockdown of *CUL4A*, *RBX1* or *SP1* caused significant repression of the proinflammatory cytokine genes, while their overexpression resulted in the reverse effects (Supplementary Figures 6A-C). In contrast, the knockdown of *NcoR1* led to significant induction of proinflammatory cytokine genes, and its overexpression caused the repression of these genes (Supplementary Figure 6D).

### PSSM0332, PSSM0856 and PSSM1437 could efficiently disrupt the CUL4A-RBX1 interaction *in vitro*

**Figure 7:**
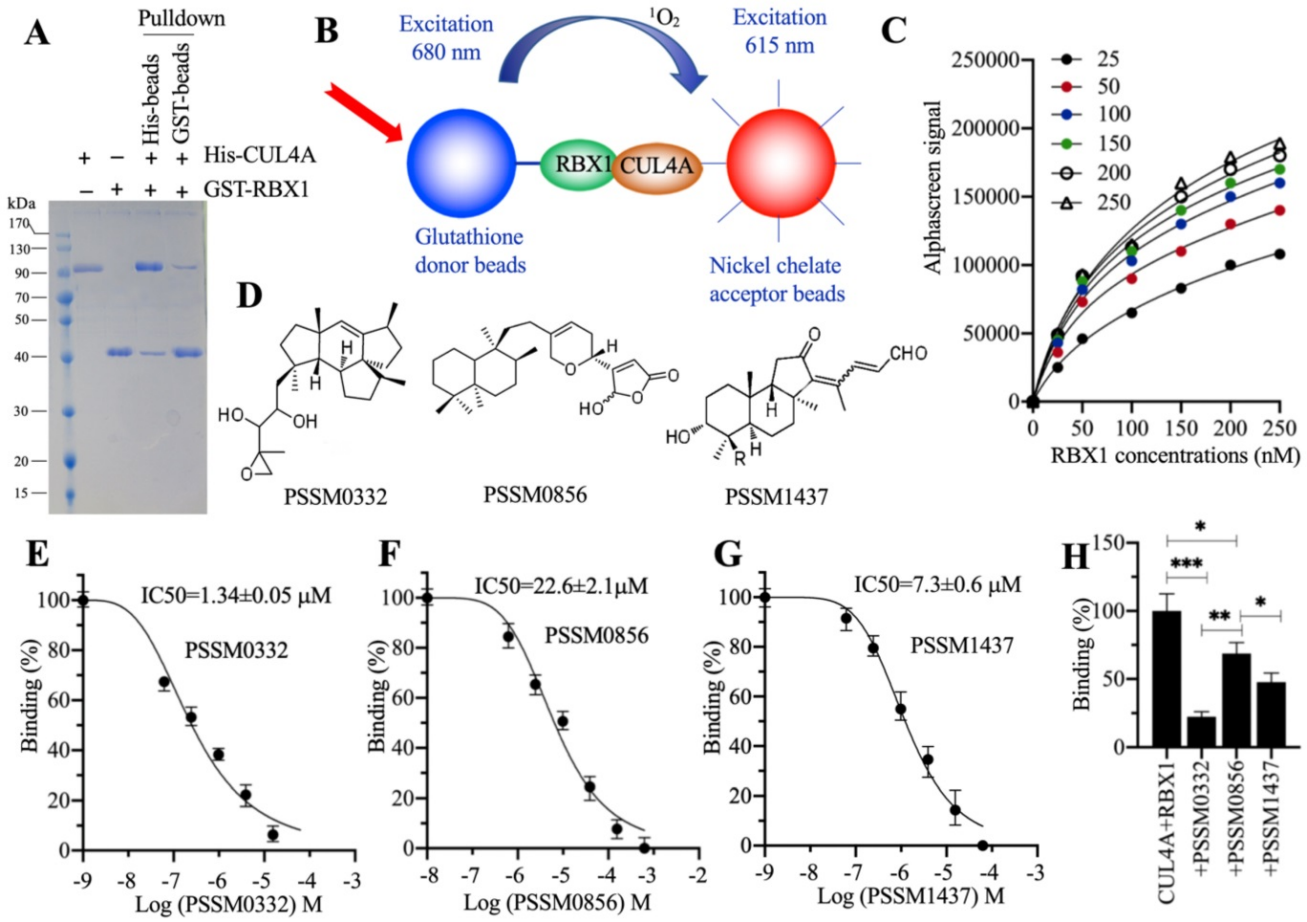
** Screening of small molecules targeting the RBX1-CUL4A interaction.** (**A**) *In vitro* pulldown result. The same concentrations (150 nM) of purified His-CUL4A and GST-RBX1 were mixed, and the protein mixture was incubated with GST beads or Ni-NTA beads. The pulldown proteins were stained with Coomassie blue. (**B**) A representative model of AlphaScreen. (**C**) The determination of optimal protein concentrations. Varying concentrations (0, 50, 100, 150, 200 and 250 nM) of GST-RBX1 were added to 25, 50, 100, 150, 200, and 250 nM His-CUL4A to generate AlphaScreen signals. (**D**) The chemical structures of PSSM0332, PSSM0856 and PSSM1437. (**E**) IC_50_ value of PSSM0332. (**F**) IC_50_ value of PSSM0856. (**G**) IC_50_ value of PSSM1437. (**H**) Comparison of the inhibitory abilities of PSSM0332, PSSM0856 and PSSM1437 at the same concentration. The same concentrations (4 µM) of three small molecules were incubated with His-CUL4A and GST-RBX1 to determine AlphaScreen signals. The signal in the mixture without small molecule supplementation was set as the control and defined as 100%, and the signals in the mixtures containing small molecules were normalized to the control. * *P* < 0.05, ** *P* < 0.01 and *** *P* < 0.001.

The conserved interactions of RBX1-CUL4A and CUL4A-DDB1 are required for the assembly of different CRL4A E3 ligases 21, 22, 25. Thus, targeting these two conserved protein interactions with small molecules should effectively disrupt the assembly of CRL4A E3 ligases. Due to the smaller molecular weight of RBX1 than DDB1, we chose RBX1-CUL4A as a target to screen small molecules in a 2500 compound pool. First, we constructed and purified His-CUL4A and GST-RBX1 (Figure 7A) and then performed *in vitro* pulldown assays using both Ni-NTA beads and GST-beads. The results showed that both His-CUL4A and GST-RBX1 could pull down each other (Figure 7A). Using these two proteins, glutathione donor beads and nickel chelate acceptor beads, we established an AlphaScreen system to screen small molecules disrupting the RBX1-CUL4A interaction (Figure 7B). For this purpose, first we determined the sensitivity and the optimal protein concentrations that were required for the AlphaScreen binding reaction. After analyzing the binding signals of different protein concentrations (Figure 7C), we selected 150 nM GST-RBX1 and 100 nM His-CUL4A to mix with donor and acceptor beads for the AlphaScreen assay. We identified three compounds (PSSM0332, PSSM0856 and PSSM1437) showing a strong ability to decrease protein binding signals (Figure 7D). Of these small molecules, PSSM0332 showed the strongest ability to disrupt the CUL4A-RBX1 interaction with an IC_50_=1.34±0.05 μM (Figure 7E), while PSSM0856 and PSSM1437 exhibited weaker abilities with IC_50_=22.6±2.1 μM (Figure 7F) and IC_50_=7.3±0.6 μM (Figure 7G), respectively. Furthermore, we also compared the inhibitory abilities of these three compounds using the same concentration (4 μM). As shown in Figure 7H, 4 μM PSSM0332 caused ~78% inhibition of the CUL4A-RBX1 interaction, while 4 μM PSSM0856 and PSSM1437 resulted in ~26% and 48% inhibition, respectively.

### PSSM0332 significantly improved the inflammatory response in SIMD mice

The promising *in vitro* inhibitory effect of PSSM0332 encouraged us to evaluate its role in improving the outcome of SIMD mice. Accordingly, we injected PSSM0332, PSSM0856 and PSSM1437 into SIMD mice at a 50 mg/kg dosage and then collected heart tissues (n=3 in each group) to examine the protein levels of the CRL4^DCAF8^ components NcoR1, SP1 and HMGB1. Our data showed that none of these small molecules could change the protein levels of CRL4^DCAF8^ components compared to those in SIMD tissues (Supplementary Figure 7A). However, the protein level of NcoR1 was significantly increased with the administration of the small molecules (Supplementary Figure 7A). In contrast, the protein levels of SP1 and HMGB1 were decreased after injecting the small molecules (Supplementary Figure 7A). Consistent with the *in vitro* results, PSSM0332 also showed the strongest improvement in changing the protein levels of NcoR1, SP1 and HMGB1 (Supplementary Figure 7A). Similar protein level patterns were also observed in the IHC staining results (Supplementary Figure 7B). Because these three small molecules were obtained based on the strategy that targeted the CUL4A-RBX1 interaction, it was not surprising that they did not change the protein levels of CRL4^DCAF8^ components. We assumed that these small molecules only affected the assembly of the CRL4^DCAF8^ E3 ligase complex, thus inhibiting the ubiquitination of NcoR1. To verify this hypothesis, we performed an *in vivo* ubiquitination assay using tissues from SIMD mice and small molecule-injected mice. As expected, our results showed that the administration of small molecules significantly decreased the ubiquitination of NcoR1 (Supplementary Figure 7C). PSSM0332 also showed the strongest inhibitory effect on NcoR1 ubiquitination, followed by PSSM1437 and PSSM0856 (Supplementary Figure 7C). In addition, we also measured the serum concentrations of proinflammatory cytokines to determine the effects of these small molecules on the inflammatory response. The ELISA results indicated that IL-1β, IL6 and TNF-α concentrations were significantly decreased in the mice injected with small molecules compared to SIMD mice (Supplementary Figure 7D-F). However, we did not observe a significant change in the concentrations of IL4 and IL13 (Supplementary Figures 8A and 8B). These results suggested that these small molecules, especially PSSM0332, might be promising candidate compounds in the therapy of SIMD.

## Discussion

**Figure 8:**
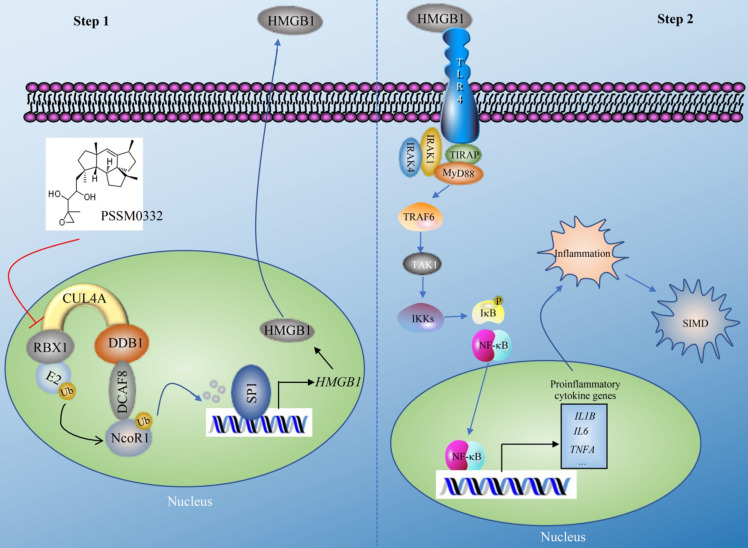
** A representative model of CRL4A^DCAF8^ E3 ligase-mediated signaling in the pathogenesis of SIMD.** CRL4A^DCAF8^ E3 ligase-mediated signaling in the pathogenesis of SIMD can be divided into two steps. In the first step, CUL4A associates with RBX1, DDB1 and DCAF8 to form the CRL4A^DCAF8^ E3 ligase complex, which recognizes NcoR1 as a substrate, leading to degradation in the pathogenesis of SIMD. The degraded NcoR1 cannot function effectively as a corepressor to inhibit SP1-mediated transcription, causing the upregulation of *HMGB1*. In the second step, mature HMGB1 in the extracellular membrane binds to TLR4 to initiate downstream events, leading to the activation of a cascade that includes TIRAP, MyD88, IRAK1, IRAK4, TRAF6, TAK1 and IKKs. The phosphorylation of IκB mediated by IKKs impairs its inhibition of NF-κB, leading to the release and translocation of NF-κB. In the nucleus, NF-κB induces the expression of proinflammatory genes and results in an inflammatory response, leading to the pathogenesis of SIMD. The small molecule PSSM0332 specifically disrupts the CUL4A-RBX1 interaction, impairing the assembly of the CRL4^DCAF8^ E3 ligase complex and affecting downstream events, eventually improving the inflammatory response and alleviating the SIMD outcome.

The CRL4 E3 ligases have been shown to have important functions in the ubiquitination of multiple proteins, thereby affecting multiple biological processes such as DNA damage and repair, cell cycle progression and tumorigenesis 21, 22. In the present study, we revealed a complex signaling controlled by CUL4A. In general, this signaling can be separated into two steps. In the first step, CUL4A assembles an E3 ligase complex with RBX1, DDB1 and DCAF8. The CRL4^DCAF8^ E3 ligase ubiquitinates and degrades NcoR1, impairing its inhibitory effect on SP1 and causing the upregulation of *HMGB1* (Figure 8). In the second step, mature HMGB1 is secreted into the extracellular membrane and functions as a cytokine to stimulate TLR4. Activated TLR4 recruits TIRAP, MyD88, IRAK1, and IRAK4 to trigger a cascade that includes TRAF6, TAK1 and IKKs. The activation of IKKs phosphorylates IκB, causing the release and activation of NF-κB, which subsequently translocates into the nucleus and induces the expression of proinflammatory genes. Mature proinflammatory cytokines induce and aggravate the inflammatory response, leading to the pathogenesis of SIMD (Figure 8). The PSSM0332 small molecule specifically targets and disrupts the CUL4A-RBX1 interaction, causing the failed assembly of the CRL4^DCAF8^ E3 ligase complex and affecting downstream events, eventually improving the inflammatory response and alleviating the SIMD outcome (Figure 8).

Inflammation is a dominant mechanism of SIMD 1, 2. However, how inflammation is initiated in the pathogenesis of SIMD is still under investigation. Using a microarray analysis, we identified a variety of differentially expressed genes, including *CUL4A* and proinflammatory cytokine genes, in SIMD heart tissues. Although we only focused our current study on *CUL4A*, the other dysregulated genes also provide valuable information for the study of SIMD pathogenesis. For example, the downregulation of *BIRC5* and the overexpression of *BAX* and *Bim* in SIMD heart tissues imply that apoptotic signaling may be activated. The activation of *HIF1A* (hypoxia-inducible factor 1-alpha) during SIMD pathogenesis suggests that a complicated transcriptional mechanism may exist to control gene expression, not only SP1-mediated signaling. The induction of multiple inflammatory response genes, such as *IL1B*, *IL6*, *TNFA*, *IFNG*, *IL12*, *IL23*, *IL33*, *IFNG*, *TGFB1*, *S100A8* and *S100A9,* suggests the successful establishment of our SIMD mouse model and supports our identification of other differentially expressed genes.

The specific overexpression of *CUL4A* but not its close paralog *CUL4B* or other cullin genes in SIMD heart tissues suggests the unique role of CUL4A in the regulation of NcoR1 ubiquitination and downstream events. Although CUL4A and CUL4B shares over 80% identify of amino acids, we did not find that downregulation or overexpression of *CUL4B* could affect *HMGB1* expression (Supplementary Figure 9). We did not investigate the underlying mechanism of *CUL4A* overexpression in this study. The result of *CUL4A* mRNA overexpression suggests that its overexpression is regulated at the transcriptional level. Several publications have shown that TFs and microRNAs are involved in the regulation of *CUL4A* or *CUL4B* in different biological processes 25, 30, 31, which provides clues for us to investigate the mechanism of *CUL4A* overexpression in the future. The conserved interactions of RBX1-CUL4 and CUL4-DDB1 are the basic scaffolds for the assembly of CRL4 E3 ligases 21, 22, 25. Using the interaction of CUL4A-DDB1 or CUL4B-DDB1 as a target, Chen et al. and Yang et al. successfully identified TSC01131 and NSC1892, respectively 24, 32. Both of these small molecules can impair the assembly of CRL4 E3 ligases during tumorigenesis 24, 32. Due to the inability to obtain these two small molecules, we could not use them as controls to examine their effects on the assembly of the CRL4A^DCAF8^ E3 ligase complex in the present study. An important issue for future studies of our three small molecules is to investigate the binding sites in RBX1-CUL4A using a protein structural strategy.

Although NcoR1 is a well-known transcriptional corepressor, it is still unknown whether it functions in the pathogenesis of SIMD. More importantly, it is also unclear whether NcoR1 can be ubiquitinated by an E3 ligase. Our finding for the first time reveals its ubiquitination and degradation by the CRL4A^DCAF8^ E3 ligase, which will significantly enhance our understanding of NcoR1 turnover during transcription. In addition, HMGB1 has previously been shown to be overexpressed in the pathogenesis of sepsis and SIMD 6, 33. However, little is known about the molecular mechanism of its overexpression in this process. A previous publication revealed that *HMGB1* and *HMGB2* can be transcriptionally regulated by NFYA in the human osteosarcoma cell line Saos-2 34. However, we did not find a change in *HMGB1* in NFYA-KD or NFYA-OE cells (Supplementary Figure 5A). The possible reason for these two different results may be because of different TFs functioning in different biological processes. Moreover, therapeutic targeting HMGB1 in sepsis animal models has shown promising results in decreasing proinflammatory cytokine levels and improving outcomes 6-8. Our results in this study suggest that targeting the upstream molecules of HMGB1 can also effectively improve SIMD outcomes in mice, which will provide more strategies for the treatment of SIMD.

In summary, we reveal a CRL4A^DCAF8^ E3 ligase-dependent ubiquitination of NcoR1, whose degradation causes the overexpression of *HMGB1*. The increased HMGB1 functions as a cytokine to trigger TLR4/NF-κB signaling, leading to inflammation and the occurrence of SIMD. The small molecule PSSM0332 can target the RBX1-CUL4A interaction and impair the assembly of CRL4A^DCAF8^ E3, thus inhibiting the ubiquitination of NcoR1, decreasing the expression of *HMGB1* and alleviating the inflammatory response. Our study reveals a complete signaling pathway that initiates the inflammatory response in the pathogenesis of SIMD and provides an effective strategy to improve the outcomes of SIMD in a mouse model, which may benefit the treatment of SIMD in the future.

## Supplementary Material

Supplementary figures and tables.
