# Supplementary material for: The small molecule PSSM0332 disassociates the CRL4ADCAF8 E3 ligase complex to decrease the ubiquitination of NcoR1 and inhibit the inflammatory response in a mouse sepsis-induced myocardial dysfunction model
Source: Int J Biol Sci. 2020 Sep 19;16(15):2974–88. doi: 10.7150/ijbs.50186 (PMC7545708; doi:10.7150/ijbs.50186)
Supplement: Supplementary file 1 — Supplementary figures and tables. [file ijbsv16p2974s1.pdf]

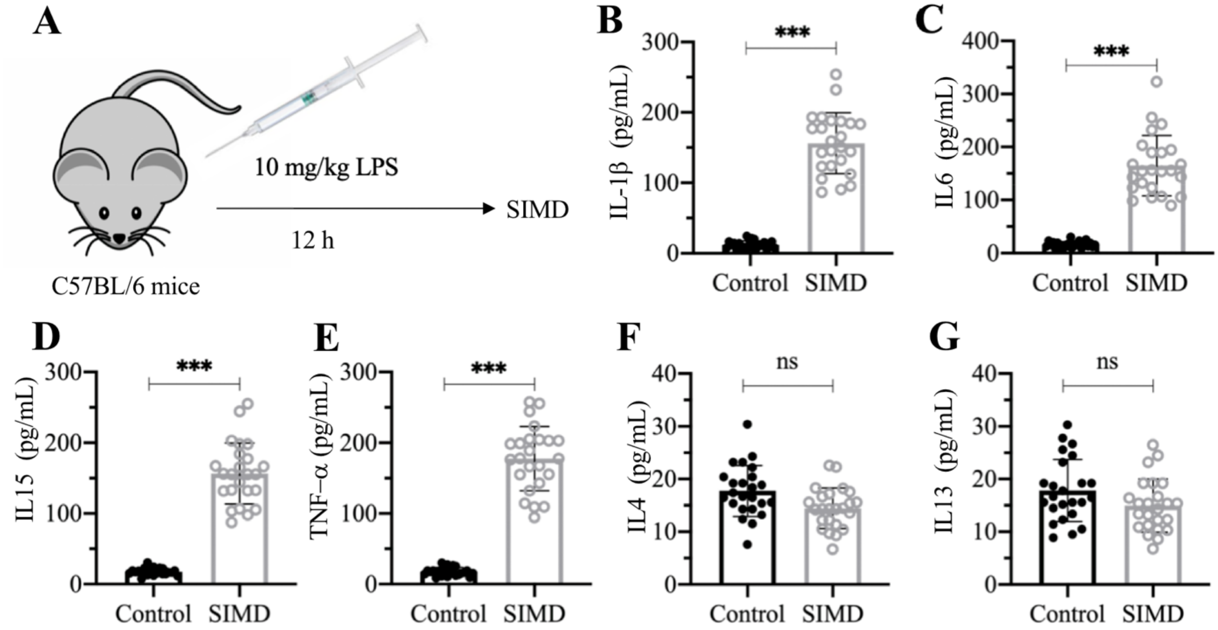

Supplementary Figure 1. The establishment of SIMD-mice and their serum concentrations of proinflammatory cytokines

(A) A representative model of LPS-induced SIMD-mouse. Similar weight (~22 g) of six-week-old C57BL/6 mice (n=48, male) were randomly divided into two groups, and were intraperitoneally injected with PBS (Control, n=24) and LPS (10 mg/kg) to generate SIMD-mice. After 12 h of injection, the blood samples were collected and the serum concentrations of four proinflammatory cytokines including IL-1β **(B)**, IL6 **(C)**, IL15 **(D)**, and TNF-α **(E)**, as well as two anti-inflammatory cytokines including IL4 **(F)** and IL13 **(G)** were examined using ELISA assays. ***P < 0.001, ns represented no significant difference.

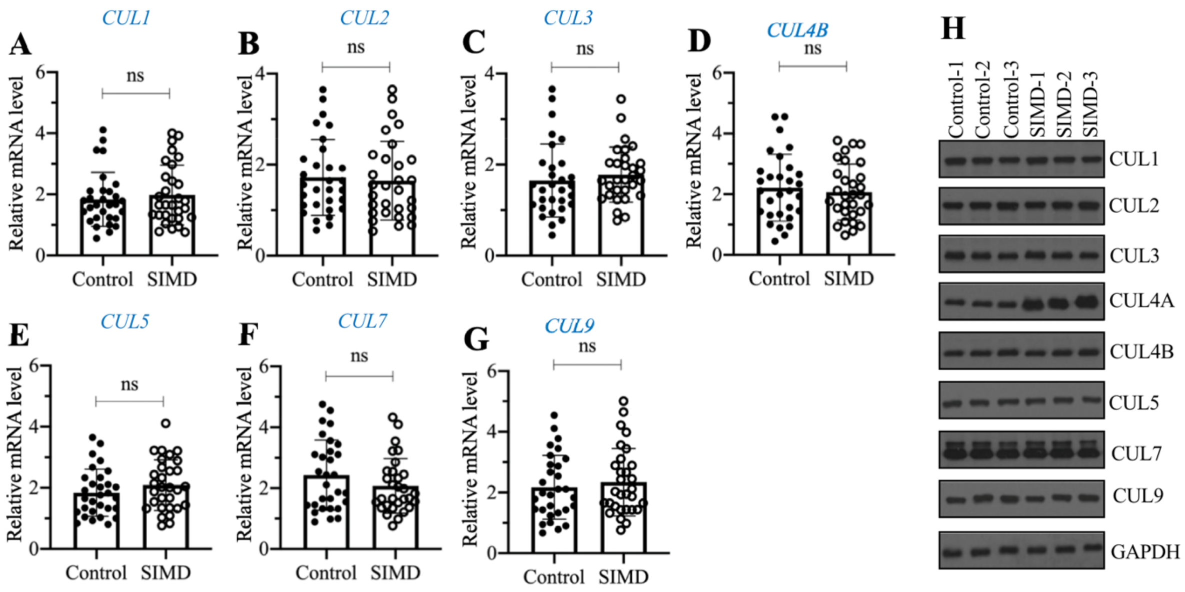

Supplementary Figure 2. The other seven cullin genes was not overexpressed in SIMD-heart tissues.

The same total RNA samples used in Figure 1B were applied to RT-qPCR analyses to examine mRNA levels of *CUL1* **(A)**, *CUL2* **(B)**, *CUL3* **(C)**, *CUL4B* **(D)**, *CUL5* **(E)**, *CUL7* **(F)**, and *CUL9* **(G).** ns represented no significant difference. **(H)** The protein levels of Cullins. Total cell extracts from three independent heart tissues of control-mice and SIMD-mice were used for western blotting to examine the protein levels of CUL1, CUL2, CUL3, CUL4A, CUL4B, CUL5, CUL7 and CUL9, respectively. GAPDH was set as a loading control.

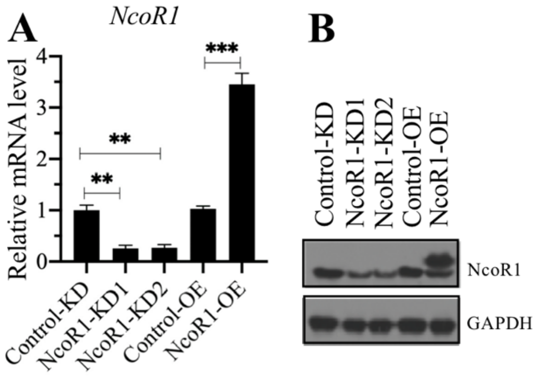

Supplementary Figure 3. The mRNA and protein levels of NcoR1 in its knockdown and overexpression cell lines.

(A) The mRNA level of *NcoR1*. Total RNA from Control-KD, NcoR1-KD1, NcoR1-KD2, Control-OE, and NcoR1-OE cells were subjected to RT-qPCR analysis to examine mRNA level of *NcoR1*. ** $P < 0.01$ and *** $P < 0.001$. **(B)** The protein level of NcoR1. Total cell extracts from cells used in (A) were subjected to immunoblots to examine NcoR1 protein level. **(C)** The relative protein level of NcoR1. The protein signals in (B) were quantified using Image J software and then normalized to their corresponding GAPDH. ** $P < 0.01$ and *** $P < 0.001$.

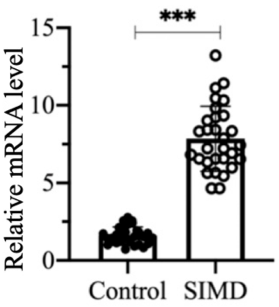

Supplementary Figure 4. The relative mRNA level of *HMGB1* in SIMD heart tissues

Total RNA samples isolated from heart tissues of controls (n=24) and SIMD mice (n=24) were used to detect the mRNA level of *HMGB1*. *** $P < 0.001$.

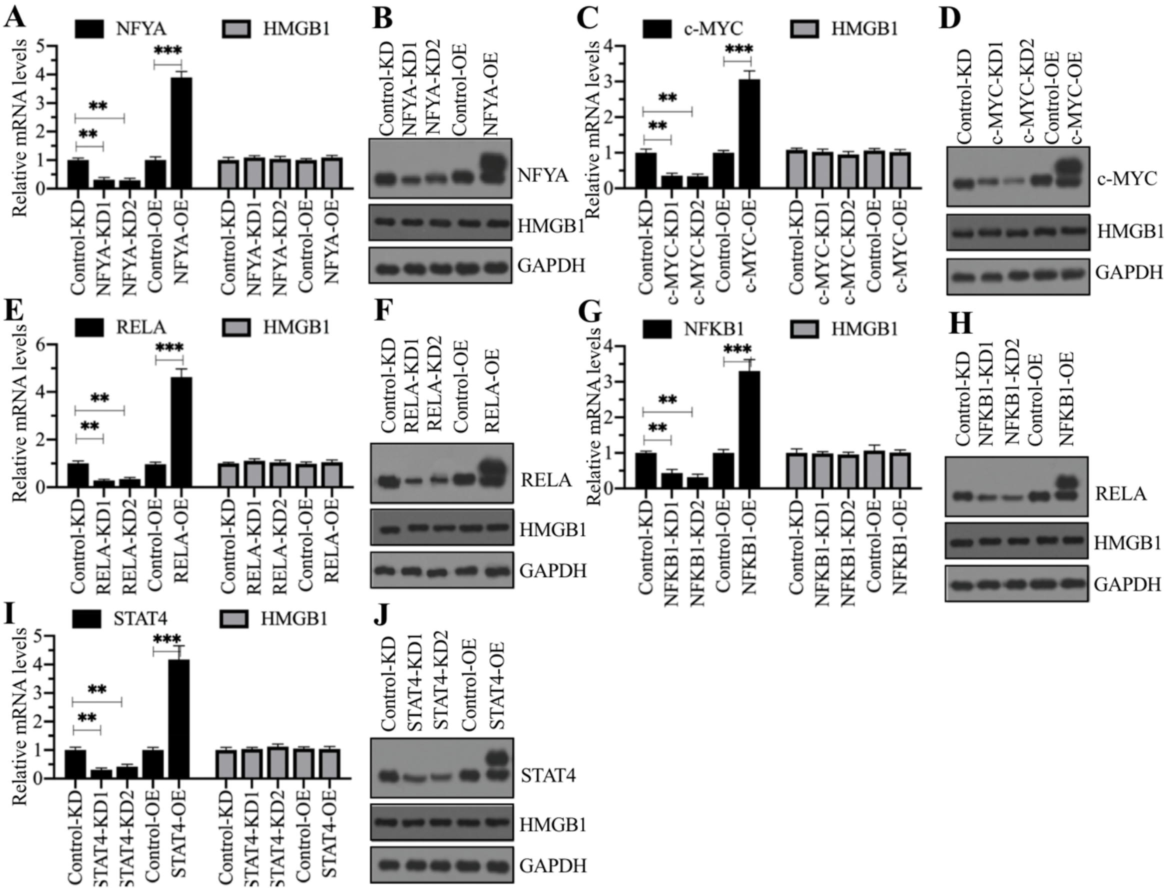

Supplementary Figure 5. The mRNA and protein levels of TFs in their corresponding knockdown and overexpression cell lines.

(A and **B)** The mRNA and protein levels of NFYA and HMGB1. Total RNA and total protein extracts from Control-KD, NFYA-KD1, NFYA-KD2, Control-OE, and NFYA-OE cells were subjected to RT-qPCR and immunoblots to examine the mRNA **(A)** and protein **(B)** levels of NFYA and HMGB1. ** $P < 0.01$ and *** $P < 0.001$. **(C** and **D)** The mRNA and protein levels of c-MYC and HMGB1. Total RNA and total protein extracts from Control-KD, c-MYC-KD1, c-MYC-KD2, Control-OE, and c-MYC-OE cells were subjected to RT-qPCR and immunoblots to examine the mRNA **(C)** and protein **(D)** levels of c-MYC and HMGB1. ** $P < 0.01$ and *** $P < 0.001$. **(E** and **F)** The mRNA and protein levels of RELA and HMGB1. Total RNA and total protein extracts from Control-KD, RELA-KD1, RELA-KD2, Control-OE, and RELA-OE cells were subjected to RT-qPCR and immunoblots to examine the mRNA **(E)** and protein **(F)** levels of RELA and HMGB1. ** $P < 0.01$ and *** $P < 0.001$. **(G** and **H)** The mRNA and protein levels of NFKB1 and HMGB1. Total RNA and total protein extracts from Control-KD, NFKB1-KD1,

NFKB1-KD2, Control-OE, and NFKB1-OE cells were subjected to RT-qPCR and immunoblots to examine the mRNA **(G)** and protein **(H)** levels of NFKB1 and HMGB1. ** $P < 0.01$ and *** $P < 0.001$. **(I** and **J)** The mRNA and protein levels of STAT4 and HMGB1. Total RNA and total protein extracts from Control-KD, STAT4-KD1, STAT4-KD2, Control-OE, and STAT4-OE cells were subjected to RT-qPCR and immunoblots to examine the mRNA **(I)** and protein **(J)** levels of STAT4 and HMGB1. ** $P < 0.01$ and *** $P < 0.001$.

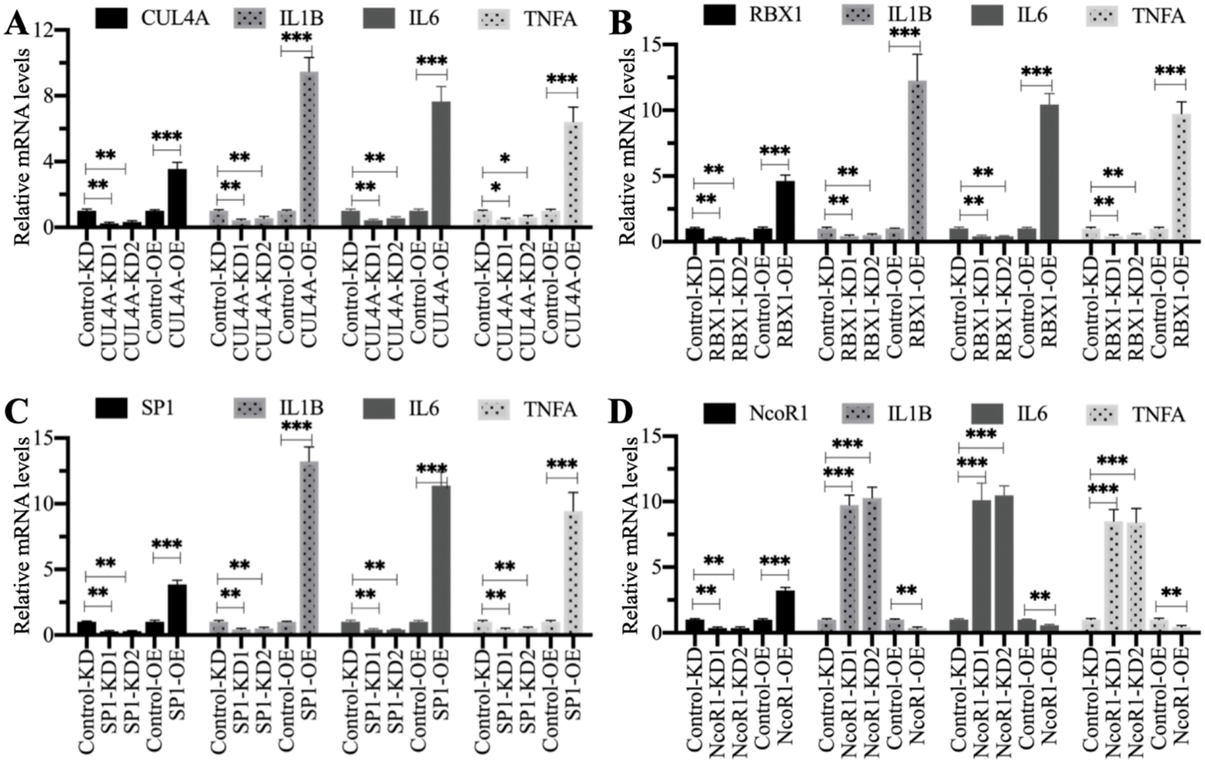

Supplementary Figure 6. The mRNA levels of proinflammatory cytokine genes in the knockdown and overexpression cells of *CUL4A*, *RBX1*, *SP1* and *NcoR1*.

(A) The mRNA levels of proinflammatory cytokine genes in the knockdown and overexpression cells of *CUL4A*. Total RNA from Control-KD, SP1-KD1, SP11-KD2, Control-OE, and SP1-OE cells were subjected to RT-qPCR analyses to examine mRNA levels of *CUL4A*, *IL1B*, *IL6* and *TNFA*, respectively. **(B)** The mRNA levels of proinflammatory cytokine genes in the knockdown and overexpression cells of *RBX1*. Total RNA from Control-KD, RBX1-KD1, RNX1-KD2, Control-OE, and RBX1-OE cells were subjected to RT-qPCR analyses to examine mRNA levels of *RBX1*, *IL1B*, *IL6* and *TNFA*, respectively. **(C)** The mRNA levels of proinflammatory cytokine genes in the knockdown and overexpression cells of *SP1*. Total RNA from Control-KD, SP1-KD1, SP1-KD2, Control-OE, and SP1-OE cells were subjected to RT-qPCR analyses to examine mRNA levels of *SP1*, *IL1B*, *IL6* and *TNFA*, respectively. **(D)** The mRNA levels of proinflammatory cytokine genes in the knockdown and overexpression cells of *NcoR1*. Total RNA from Control-KD, NcoR1-KD1, NcoR1-KD2, Control-OE, and NcoR1-OE cells were subjected to RT-qPCR analyses to examine mRNA levels of *NcoR1*, *IL1B*, *IL6* and *TNFA*, respectively.

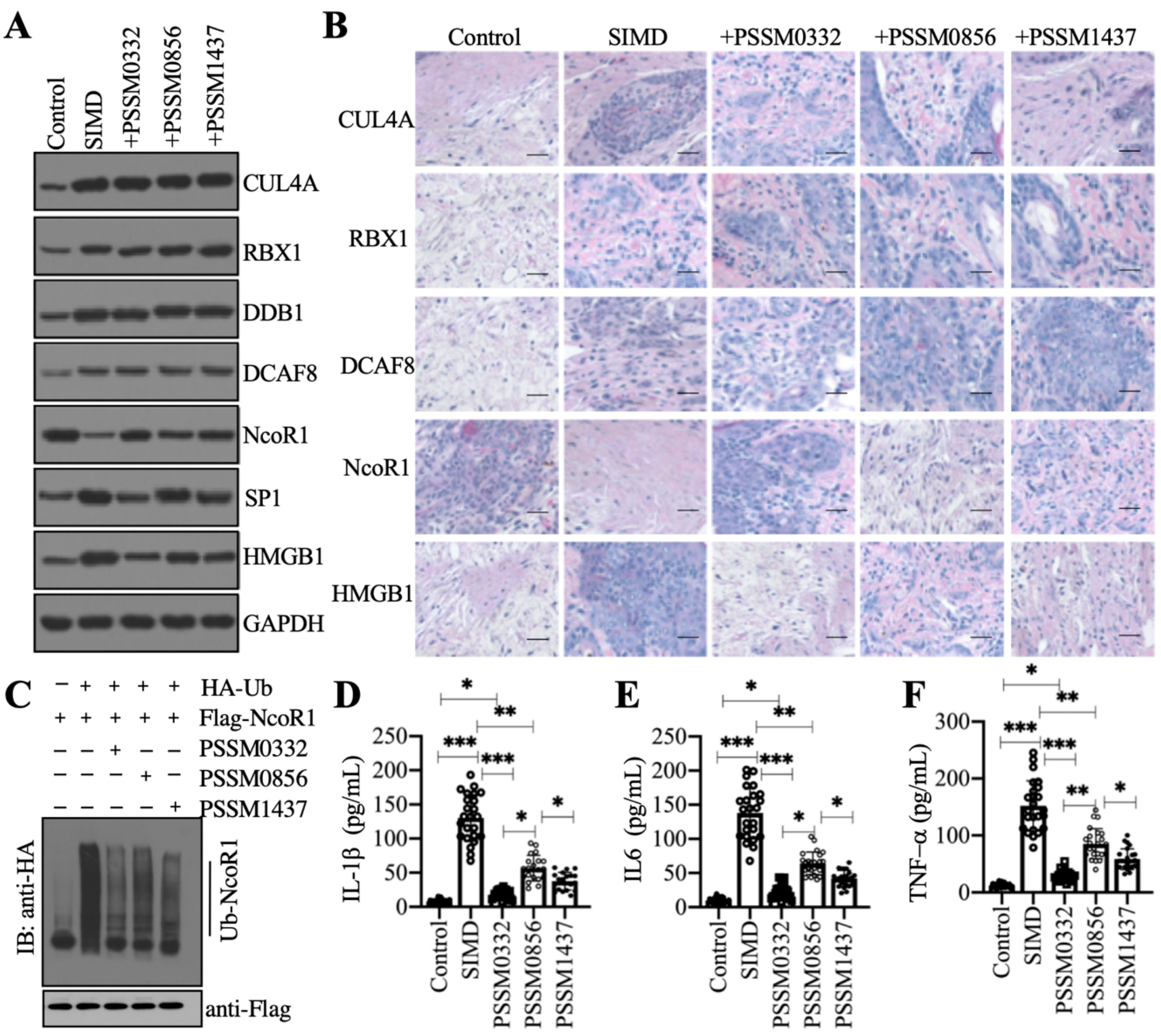

Supplementary Figure 7. PSSM0332 significantly reversed the CRL4A^{DCAF8} downstream events.

(A) The effects of PSSM0332 on the protein levels of the CRL4A^{DCAF8} components NcoR1, SP1 and HMGB1. Three independent heart tissues from the untreated mice (control), SIMD mice, and SIMD mice injected with PSSM0332, PSSM0856 or PSSM1437 were subjected to protein isolation and immunoblots to examine the protein levels of CUL4A, RBX1, DDB1, DCAF8, NcoR1, SP1 and HMGB1. GAPDH was used as a loading control. **(B)** Representative IHC staining images of heart tissues. Three independent heart tissues from the untreated mice (control), SIMD mice, and SIMD mice injected with PSSM0332, PSSM0856 or PSSM1437 were subjected to IHC staining with anti-CUL4A, anti-RBX1, anti-DCAF8, anti-NcoR1 and anti-HMGB1, respectively. A representative image from each group is presented. Bars= 100 μm. **(C)** *In vivo* ubiquitination assay results. RAW246.7 cells were transfected with pcDNA3-2×Flag-NcoR1 alone or

cotransfected with pcDNA3-2×Flag-NcoR1 and HA-ubiquitin plasmids. After 48 h, the cells cotransfected with pcDNA3-2×Flag-NcoR1 and HA-ubiquitin were further treated with 4 μM PSSM0332, PSSM0856 or PSSM1437 for 6 h. Cells were immunoprecipitated with an anti-Flag antibody, and the ubiquitination of NcoR1 was detected using an anti-HA antibody. ^{Flag}NcoR1 was a loading control. **(D-F)** The serum concentrations of proinflammatory cytokines. Blood samples from untreated mice (Control), SIMD mice, and SIMD mice injected with PSSM0332, PSSM0856 or PSSM1437 were used for ELISAs to measure the circulating levels of IL-1β **(D)**, IL6 **(E)** and TNF-α **(F)**. * $P < 0.05$, ** $P < 0.01$ and *** $P < 0.001$.

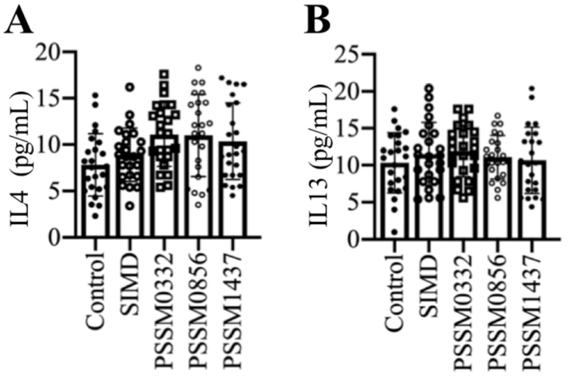

Supplementary Figure 8. The serum concentrations of IL4 and IL13 in SIMD-mice treated with small molecules.

The blood samples used in Supplementary Figure 7D were subjected to ELISA assays to measure the concentrations of IL4 **(A)** and IL13 **(B)**.

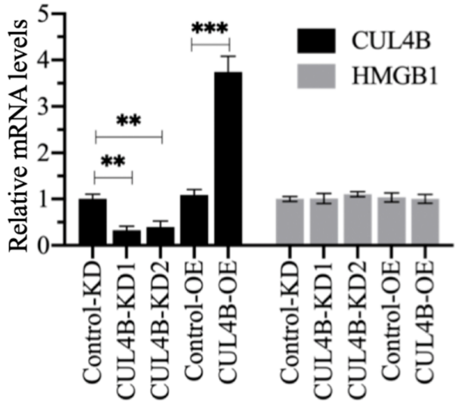

Supplementary Figure 9. The relative mRNA levels of *CUL4B* and *HMGB1*

Total RNA samples isolated from Control-KD, CUL4B-KD (#1 and #2), Control-OE, and CUL4B-OE cells were used to detect the mRNA level of *CUL4B* and *HMGB1*. ** $P < 0.01$ and *** $P < 0.001$.

Supplementary Table-1. Primers used for RT-qPCR analyses

Gene	Forward Primers	Reverse primers
CUL1	5'-GGAAGGTCCTGAAACACCAG-3'	5'-CTTCTCACCATCGACTCGCTC-3'
CUL2	5′-TCGCTGGCACCCTTCCACAGT-3′	5'-ACCAGAGTATCTGAATGCC-3'
CUL3	5'-GTGAAGACAGCTCAGCTGTA-3'	5'-GTAAGGTAGCAGAGCCAGCA-3'
CUL4A	5'-AGGCTGCCACTCGGATCTCTGC-3'	5'-GTCTCATGACAGACATGATGT-3'
CUL4B	5'-ATCAAGCATGCTACTGGGAT-3'	5'-GTCACCATCTTCAATATCT-3'
CUL5	5'-GGAGCAGATGGAGTGGCTG-3'	5'-AGCCTCTGCCCAGGCCTTCAG-3'
CUL7	5'-CCTTGTTGTCCGAATCCTC-3'	5'-ATCAGAGCTCCTGCAGGTT-3'
CUL9	5'-GAGTACATGGATGTGGTAG-3'	5'-TGAGGCAGTCGGCCCGCAGGAA-3'
IL1B	5'-AGTTCCCCAACTGGTACAT-3'	5'-CTGGGGAAGGCATTAGAAACAGT-3'
S100A8	5'- TCGTGACAATGCCGTCTGAACT-3'	5'-CTGCACAAACTGAGGACACTCA-3'
PLD2	5'-AGCTGTGGCAAGAAACAGCGGA-3'	5'-GCAAGCTCAGACTGAGCCAAA-3'
ZFP91	5'-AGGCTTACCGTACCTTCCAAGG-3'	5'-TCCTCTAGCTGGTAGGCGAA-3'
BIRC5	5'-GATGACAACCCGATAGAGGA-3'	5'-TGTTGTTGGTCTCCTTTGCA-3'
NcoR1	5'-ACGGATGCTCAGCAGTACACC-3'	5'-TCAGTCGTCACTATCAGACA-3'
HMGB1	5′-TCCTTACACGAGGACTCTCCT-3′	5'-CGCTGGGACTAAGGTCAACA-3'
IL6	5′-GCTGGAGTCACAGAAGGAGTG-3′	5'-CACAGTGAGGAATGTCCAC-3'
CCL2	5′-CTACAAGAGGATCACCAGCAG-3′	5'-TGTCTGGACCCATTCCTTC-3'
DNM2	5′-ATGGAGCACAAGAGAACAC-3′	5'-TCATGAGGTGCATGATGGTC-3'
FEN1	5′-CAGCAAGTACCCCGTTCCAGA-3′	5'-TCTTCTTCATTTGGCTCGCT-3'
CCN2	5′-CTACAAGAGGATCACCAGCA-3′	5'-TGATCTCATTTGGTTCCGATC-3'
NFYA	5′-TCTAGAGAGCAAGGGCCCTG-3′	5'-CTTCAGGCAATATAAGGAT-3'
c-MYC	5′-TCCAGGACTGTATGTGGAG-3′	5'-GGTGAGGTCCTGCAGGTACA-3'
RELA	5′-TGCTGATGGAGTACCCTGA-3′	5'-AGTCCATGTCCGCAATGGA-3'
NFKB1	5′-AATGGGAAACCGTATGAG-3′	5'-GTGGCCCAGTTTTTGTCTG-3'
STAT4	5′-TCTAATGTCAGCCAACT-3′	5'-GACATAGGATGAAAATTGCC-3'
SP1	5′-TCTCCACAGCACTCATGTGAG-3′	5'-CAGGCTCCTCTACCTCCAC-3'
β-Actin	5'-TACAGCTTCACCACCACAGC-3′	5'- GGCAGCTCATAGCTCTTCT -3'

Supplementary Table-2. The aberrantly expressed genes in SIMD-heart tissues

Gene	Gene description	Average fold change	P Value	Expression
PLD2	Phospholipase D2	-18.2	0.00073	Down
ALAS1	5'-Aminolevulinate Synthase 1	-16.3	0.0021	Down
TGM2	Transglutaminase 2	-14.5	0.0056	Down
LDHA	Lactate Dehydrogenase A	-13.2		Down
ALDH1A1	Aldehyde Dehydrogenase 1 Family Member A1	-11.8	0.00092	Down
PTEN	Phosphatase And Tensin Homolog	-10.2	0.000051	Down
TAF5	TATA-Box Binding Protein Associated Factor 5	-9.8	0.00088	Down
WSB1	WD Repeat And SOCS Box Containing 1	-9.2	0.00035	Down
GCAT	Glycine C-Acetyltransferase	-9.8	0.00092	Down
CIAO1	Cytosolic Iron-Sulfur Assembly Component 1	-9.3	0.00078	Down
ZFP91	Zinc Finger Protein 91	-9.1	0.00088	Down
GNG11	G Protein Subunit Gamma 11	-8.3	0.0032	Down
XIAP	X-Linked Inhibitor Of Apoptosis	-7.6	0.0082	Down
BARD1	BRCA1 Associated RING Domain 1	-7.1	0.0012	Down
NR1H3	Nuclear Receptor Subfamily 1 Group H Member 3	-6.6	0.00045	Down
PGS1	Phosphatidylglycerophosphate Synthase 1	-5.3	0.00035	Down
CDH1	Cadherin 1	-4.6	0.00094	Down
BIRC5	Baculoviral IAP Repeat Containing 5	-4.2	0.00074	Down
PIAS3	Protein Inhibitor Of Activated STAT3	-3.3	0.00083	Down
IL1B	Interleukin 1 beta	19.4	0.00071	Up
HMGB1	High Mobility Group Box 1	17.5	0.00054	Up
IL6	Interleukin 6	16.4	0.000092	Up
TNFA	Tumor Necrosis Factor-Alpha	15.4	0.0022	Up
CUL4A	Cullin 4A	14.6	0.0018	Up
IL15	Interleukin 15	13.8	0.0032	Up
IL18	Interleukin 18	12.6	0.0036	Up
IFNG	Interferon Gamma	11.5	0.0044	Up
CSF1	Colony Stimulating Factor 1	10.9	0.0019	Up
IL12	Interleukin 12	10.2	0.00048	Up
BAX	BCL2 Associated X	9.6	0.00038	Up
Bim	Bcl-2 Interacting Mediator	9.2	0.00044	Up
MMP2	Matrix Metallopeptidase 2	8.7	0.00085	Up
NOD2	Nucleotide Binding Oligomerization Domain Containing 2	8.2	0.00029	Up
SP1	Specificity Protein 1	7.6	0.0012	Up
NOS1	Nitric Oxide Synthase 1	7.2	0.0044	Up
ICAM1	Intercellular Adhesion Molecule 1	6.8	0.0065	Up
TLR4	Toll Like Receptor 4	6.2	0.0019	Up
TGFB1	Transforming Growth Factor Beta 1	5.6	0.0043	Up
ITGA8	Integrin Subunit Alpha 8	5.2	0.0012	Up
HIF1A	Hypoxia Inducible Factor 1 Subunit Alpha	4.7	0.0055	Up
IL33	Interleukin 33	4.3	0.0012	Up
IL23	Interleukin 23	3.8	0.00093	Up

PRF1	Perforin 1	3.2	0.00012	Up
S100A8	S100 Calcium Binding Protein A8	3.1	0.00032	Up
S100A9	S100 Calcium Binding Protein A9	2.6	0.00077	Up

Supplementary Table-3. The DDB1-associated proteins identified by LC-MS/MS

Protein	Protein description	Molecular weight (kDa)	MASCOT scores
DDB1	Damage Specific DNA Binding Protein 1	127	1092
DCAF8	DDB1 And CUL4 Associated Factor 8	67	984
CUL4A	Cullin 4A	88	901
RBX1	RING box 1	12	832
SIRT7	Sirtuin 7	45	798
PCID2	PCI Domain Containing 2	46	722
DDA1	DET1 And DDB1 Associated 1	12	674
GLMN	Glomulin, FKBP Associated Protein	68	602
GRWD1	Glutamate Rich WD Repeat Containing 1	49	573
CDT1	Chromatin Licensing And DNA Replication Factor 1	60	552
COPS8	COP9 Signalosome Subunit 8	23	489
KEAP1	Kelch Like ECH Associated Protein 1	70	431
SPOP	Speckle Type BTB/POZ Protein	42	409
b-Actin	Actin Beta	42	387
KCTD10	Potassium Channel Tetramerization Domain Containing 10	35	325
WSB1	WD repeat and SOCS box containing 1	47	321
NEDD8	NEDD8 Ubiquitin Like Modifier	9	303
SP1	Specificity Protein 1	81	296
ITCH	Itchy E3 Ubiquitin protein ligase	103	287
ERCC8	ERCC Excision Repair 8, CSA Ubiquitin Ligase Complex Subunit	44	277
NcoR1	Nuclear Receptor Corepressor 1	270	263
DCUN1D2	Defective In Cullin Neddylation 1 Domain Containing 2	30	208

Supplementary Table-4. **The DCAF8-associated proteins identified by LC-MS/MS**

Protein	Protein description	Molecular weight (kDa)	MASCOT scores
DCAF8	DDB1 And CUL4 Associated Factor 8	67	1523
DDB1	Damage Specific DNA Binding Protein 1	127	1435
CUL4A	Cullin 4A	88	1409
RBX1	RING box 1	12	1334
GAN	Gigaxonin	68	1284
VIM	Vimentin	54	1209
ACTG1	Actin Gamma 1	42	1134
ARHGEF1	Rho Guanine Nucleotide Exchange Factor 1	103	1008
ANKRD36	Ankyrin Repeat Domain 36	217	993
PEX19	Peroxisomal Biogenesis Factor 19	33	942
NCSTN	Nicastrin	78	846
CASQ1	Calsequestrin 1	45	804
IGSF8	Immunoglobulin Superfamily Member 8	65	779
CDT1	Chromatin Licensing And DNA Replication Factor 1	60	735
DCUN1D2	Defective In Cullin Neddylation 1 Domain Containing 2	30	665
COPS8	COP9 Signalosome Subunit 8	23	626
KPNA1	Karyopherin Subunit Alpha 1	66	598
WSB1	WD repeat and SOCS box containing 1	47	556
WDTC1	WD And Tetratricopeptide Repeats 1	76	524
PPOX	Protoporphyrinogen Oxidase	51	467
SLAMF6	SLAM Family Member 6	37	433
β-Actin	Actin Beta	42	386
USP21	Ubiquitin Specific Peptidase 21	63	365
GBP2	Guanylate binding protein 2	67	343
DEDD	Death Effector Domain Containing	37	324
PRMT1	Protein Arginine Methyltransferase 1	42	309
TAGLN2	Transgelin 2	22	302
AMPD2	Adenosine Monophosphate Deaminase 2	101	289
PDE7A	Phosphodiesterase 7A	56	277
CTRL1	Chymotrypsin-like protease	28	265
NcoR1	Nuclear Receptor Corepressor 1	270	254
DTL	Denticleless E3 Ubiquitin Protein Ligase Homolog	79	235
TNS2	Tensin 2	153	218
ITLN1	Intelectin 1	35	209

Supplementary Table-5. The aberrantly expressed dependent on NcoR1

Gene	Gene description	NcoR1-KD	NcoR1-OE
HMGB1	High Mobility Group Box 1	10.3	-8.6
IL1B	Interleukin 1 beta	9.8	-8.2
TNFA	Tumor Necrosis Factor-Alpha	8.4	-7.8
IL6	Interleukin 6	8.2	-8.4
IL15	Interleukin 15	7.6	-6.8
IL18	Prostate cancer associated transcript 6	7.1	-8.1
CCL3	C-C Motif Chemokine Ligand 3	6.4	-6.1
CCL6	C-C Motif Chemokine Ligand 6	5.4	-7.2
CXCL12	C-X-C Motif Chemokine Ligand 12	5.1	-5.5
IFNG	Interferon Gamma	4.5	-6.4
ITGAV	Integrin Subunit Alpha V	4.2	-4.6
S100A12	S100 Calcium Binding Protein A12	3.8	-5.6
CBX3	Chromobox 3	3.1	-3.8
SP100	Speckled 100 KDa	2.5	-4.5
CCNA2	Cyclin A2	2.2	-5.3
IFNB1	Interferon Beta 1	5.2	-3.4
MSH2	MutS Homolog 2	4.3	-4.6
CCL2	C-C Motif Chemokine Ligand 2	3.6	-3.9
MECP2	Methyl-CpG Binding Protein 2	3.1	-4.5
BRD4	Bromodomain Containing 4	2.8	-3.6
DNM2	Dynamin 2	-11.3	9.3
RFC1	Replication Factor C Subunit 1	-9.6	7.4
ZC3H12A	inc Finger CCCH-Type Containing 12A	-8.4	8.2
FEN1	Flap Structure-Specific Endonuclease 1	-8.1	5.6
PLG	Plasminogen	-7.6	8.3
CMA1	Chymase 1	-6.5	4.5
SOX18	SRY-Box Transcription Factor 18	-5.4	7.2
CTNNB1	Catenin Beta 1	-4.8	5.4
CCN2	Cellular Communication Network Factor 2	-4.6	3.2
